# Acyclic Hydrocarbon *α*,*ω*‐Dienes: From Biomass and Plastic Upcycling to Carbocycles, Precision Polymers, and Cross‐Linked Elastomers

**DOI:** 10.1002/cssc.70848

**Published:** 2026-07-11

**Authors:** Rosa Villaccio, Giuseppe Leone

**Affiliations:** ^1^ CNR‐Istituto di Scienze e Tecnologie Chimiche “Giulio Natta” (SCITEC) Milano Italy

**Keywords:** *α*,*ω*‐dienes, carbocycles, elastomers, insertion polymerization, plastic upcycling

## Abstract

Acyclic hydrocarbon *α*,*ω*‐dienes constitute a large class of organic molecules featuring two C=C bonds separated by methylene segments of variable length and with an open, linear chain. *α*,*ω*‐Dienes serve as substrates and (co)monomers in a range of key chemical transformations. These include insertion (cyclo)polymerization, metathesis, cyclization, and hydroformylation to yield chemicals and polymers with potential applications in adhesives, lubricants, impact modifiers, automotive, membranes, and pharmaceuticals. The production of *α*,*ω*‐dienes currently relies on the conversion of steam‐cracking derivatives; however, the high cost, operational complexity, and substantial environmental burden associated to these technologies restrain their use to niche academic studies. The first section of this review provides a concise snapshot of emerging technologies for the production of such *α*,*ω*‐dienes from biomass‐derived feedstocks and via chemical plastic upcycling. These, alternative to petrochemicals, are critical to meet the pressing sustainability goals and to ensure the long‐term accessibility of *α*,*ω*‐dienes. The second and main section surveys the catalytic conversion of *α*,*ω*‐dienes into carbocycles, (di)aldehydes, precision unsaturated poly(ethylene)s and elastomers. *α*,*ω*‐Dienes act as modular, programmable molecular “Lego” blocks to access carbocycles, from strained rings to macrocycles, and to polymers with tailored functionalities and strategically placed cleavable sites that facilitate reuse, recycling, deconstruction, and predictable end‐of‐life behavior.

## Graphical abstract

Hydrocarbon *α*,*ω*‐dienes are mainly produced from steam‐cracking derivatives, but high costs, complexity, and environmental impact limit their use. This review highlights sustainable paths from biomass and plastic upcycling, and surveys catalytic transformations into carbocycles and specialty polymers, emphasizing their role as modular building blocks for advanced materials and rings.

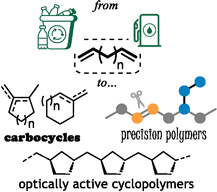

## 1 Introduction and Scope of the Review

Non‐conjugated *α*,*ω*‐dienes are a large class of organic molecules characterized by the presence of two C=C bonds separated by methylene segments of variable length. Acyclic, hydrocarbon *α*,*ω*‐dienes, for example, 1,5‐hexadiene, 1,7‐octadiene and 1,9‐decadiene, are the most structurally simple subclass of non‐conjugated dienes, composed solely of carbon and hydrogen atoms and defined by an open, linear chain. The *α*,*ω* designation indicates that the two C=C bonds are positioned at the terminal ends of the carbon chain. This arrangement ensures that the terminal vinyls are sufficiently isolated to prevent electronic interaction, as observed in conjugated 1,3‐dienes, while facilitating selective and orthogonal functionalization, as well as diverse intramolecular and intermolecular transformations.

Acyclic *α*,*ω*‐dienes are extensively employed as (co)monomers for step‐growth and chain‐growth polymerization, including insertion (cyclo)polymerization [1], acyclic diene metathesis (ADMET) [2, 3], and ring‐closing metathesis (RCM) [4], to yield precision unsaturated poly(ethylene)s (PEs), (cyclo)polymers and elastomers. *α*,*ω*‐Dienes are also effective intermediates to generate cyclic compounds [5], from strained 3‐ and 4‐membered rings to macrocyclics, via cyclization, cycloisomerization, and cycloaddition; these motifs are key structural elements in natural products and pharmaceutical molecules. However, synthesis of linear hydrocarbon *α*,*ω*‐dienes largely relies on the conversion of fossil‐derived hydrocarbons obtained via steam cracking and typically involves specialized, costly multistep reactions, and precious transition metal catalysts [6]. As a consequence, their availability and cost are tightly associated with fossil carbon supply chains, raising concerns regarding sustainability, price volatility, and environmental impact. In contrast to conjugated dienes, such as 1,3‐butadiene and isoprene, which are abundant and readily available commodities [7], acyclic *α*,*ω*‐dienes have historically received comparatively less attention in both academic and industrial research. The high cost, operational complexity and substantial environmental burden associated with current supply methodologies restrict the application of *α*,*ω*‐dienes to niche markets and academic studies.

The 20th century witnessed the emergence of more sustainable and economically viable strategies to produce *α*,*ω*‐dienes. Advances in the upgrading of biomass‐derived molecules and in plastic chemical upcycling have renewed interest in *α*,*ω*‐diene chemistry and grant milder and more selective alternatives. Greater availability and ready access to *α*,*ω*‐dienes would not only mitigate environmental impact but also stimulate further exploration of *α*,*ω*‐diene reactivity. In turn, this could unlock new opportunities and applications for such simple hydrocarbons across materials science, organic electronics, membrane technologies and green chemistry. This review highlights recent advances in the sourcing of hydrocarbon *α*,*ω*‐dienes from chemical upcycling of commodity plastic waste, as well as their downstream conversion into specialty and precision polymers via insertion (co)polymerization and post‐polymerization modification. Emphasis is placed on how the monomer size (and substituents), and the metal (pre)catalyst choice govern polymerizability, reactivity and selectivity. The review is intended as a primer for readers less familiar with *α*,*ω*‐diene enchainment in chain‐growth polymerization, whereas it also offers practical and mechanistic discussions for experienced olefin insertion polymerization practitioners. We begin with a short historical and mechanistic overview, followed by a discussion of the polymer properties, potential real‐world applications and selectivity, integrating, where available, insights from both experimental and computational studies. To set the scene, a short synopsis of synthetic paths to access *α*,*ω*‐dienes from biomass, either as the primary or valuable co‐products, is provided. Subsequent sections examine the use of *α*,*ω*‐dienes in ADMET, cyclization, cycloaddition, RCM, and hydroformylation to access unsaturated polymers with cleavable linkages, carbocycles and (di)aldehydes. This domain is so vast, encompassing hundreds of functionalized *α*,*ω*‐dienes and reaction conditions, that it is nontrivial to appropriately select the required reaction conditions [6, 8, 9]. Accordingly, this section provides only a critical analysis of the literature rather than a comprehensive catalog of examples. The goal is to explore the evolution and current landscape of such key transformations and to offer a practical guide for selecting appropriate dienes, reagents, and catalysts for specific reactions.

The ultimate goal is to outline emerging opportunities and to identify critical challenges, including issues of scalability, stability, productivity and operational simplicity associated with hydrocarbon non‐conjugated diene sourcing and valorization.

## 2 Acyclic Non‐Conjugated *α*,*ω*‐Dienes

### 2.1 Current Supply From 1,3‐Dienes and Olefins

**FIGURE 1:**
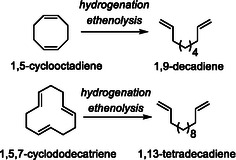
Examples of synthesis of *α*,*ω*‐dienes from dimerization and trimerization of 1,3‐butadiene.

The most established approaches for synthesizing high‐purity acyclic hydrocarbon *α*,*ω*‐dienes rely on the conversion of steam‐cracking derivatives (i.e., ethylene, 1,3‐butadiene, or allylic halides). Such technologies typically involve cost‐intensive multistep reactions, precious transition metal catalysts, non‐benign reagents, and intensive energy consumption to selectively access the desired isolated double bond structure [10, 11, 12, 13]. A representative example is the dimerization and trimerization of 1,3‐butadiene which affords a mixture of 1,5‐cyclooctadiene and 1,5,7‐cyclododecatriene. Partial hydrogenation of 1,5‐cyclooctadiene followed by ethenolysis affords the desired 1,9‐decadiene [14, 15]; similarly, 1,13‐tetradecadiene can be accessed from 1,5,7‐cyclododecatriene using the same reaction sequence (Figure 1). Ethenolysis of 1,5,7‐cyclododecatriene generates 1,5‐hexadiene [15]. Overall, the significant expense, operational complexity and substantial environmental burden associated with such methodologies currently restrict the industrial application of *α*,*ω*‐dienes to niche markets. Consistent with these limitations, large companies such as Shell, which previously produced 1,9‐decadiene from cyclooctene and ethylene, or Chemische Werke Hüls (now part of Evonik) which produced 1,7‐octadiene by a bimolecular catalytic reduction process of 1,3‐butadiene promoted by HCOOH/NEt_3_ [16], no longer produce these chemicals.

**FIGURE 2:**
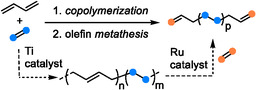
Schematic illustration of two‐step *α*,*ω*‐diene generation proposed by Agapie et al. [17].

Recently, Agapie et al. reported a two‐step process to generate a distribution of *α*,*ω*‐dienes from upcycling of a purpose‐designed copolymer from ethylene and 1,3‐butadiene [17]. In the first step, the copolymer with randomly distributed internal C=C bonds was synthesized using a Ti bisphenoxide bisthiolate catalyst. In the second step, olefin metathesis using a Ru catalyst yields a distribution of *α*,*ω*‐dienes in the C10–C20 range as the major species (Figure 2). In addition to *α*,*ω*‐dienes, telechelic polyolefins and small amounts of odd‐carbon‐number *α*‐olefins (less accessible and more expensive than even‐carbon counterparts produced by the commercial Shell higher olefin process) are also obtained.

### 2.2 Future Trajectory: From Biomass to Plastic Waste Upcycling

The future trajectory of hydrocarbon *α*,*ω*‐dienes will depend significantly on addressing the pressing challenges of sustainability. The most promising and desirable technologies for the sustainable production of *α*,*ω*‐dienes are those that leverage biomass‐derived feedstocks and the chemical upcycling of plastic waste [18, 19, 20, 21]. Such a transition aligns with global sustainability targets, and it is a key step in the decarbonization of the chemical industry through the integration of renewable feedstocks and advanced recycling strategies.

Renewable feedstocks, including furfural, 5‐hydroxymethylfurfural, fatty acids, esters from vegetable oils or microalgae and diols may be a compelling source for selective synthesis of *α*,*ω*‐dienes. Yet, current technologies are far from achieving this goal. As an example, dehydration of linear diols to *α*,*ω*‐dienes is often limited by low selectivity and competition from side reactions, including C—C bond cleavage, deoxygenation, and other secondary processes [22, 23]. However, Zhou, Chen et al. reported an efficient aliphatic‐acid‐mediated dehydration of C6–10‐*α*,*ω*‐alkanediols to alkadienes. The conversion proceeds in a stepwise manner: alkanediols react with aliphatic acids first to generate diesters; subsequent pyrolysis of the latter produces alka‐*α*,*ω*‐dienes. The highest yields of 1,5‐hexadiene, 1,7‐octadiene, and 1,9‐decadiene were up to 70.3%, 74.8%, and 90.3%, respectively [23]. Lei, Chen et al. demonstrated that 1,5‐hexadiene is generated as a secondary product during the chemical transformation of bio‐based ethanol to styrene, along with several other by‐products [24]. Interestingly, sensitivity analysis identifies 1,5‐hexadiene as the by‐product with the strongest impact on the minimum product selling price. This is because a fraction of 1,5‐hexadiene is combusted to supply heat, while the remaining fraction is recovered and sold. As a result, its allocation directly influences both economic and environmental performance. The authors estimate that, assuming a market price of $80,000 per ton, an optimal balance between these metrics is achieved at combustion ratios between 0.65 and 0.75. These findings highlight the critical role of 1,5‐hexadiene management in determining the overall efficiency and sustainability of the bioethanol upgrading process.

**FIGURE 3:**
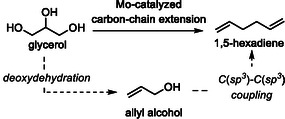
Glycerol upgrading to 1,5‐hexadiene [25].

In 2025, Lu et al. reported a novel, efficient and greener route to produce 1,5‐hexadiene from biomass‐derived glycerol, using a non‐noble Mo complex [25]. The methodology successfully converts glycerol into 1,5‐hexadiene (yield = 52%) using a Mo complex with an 8‐hydroxyquinoline ligand and in the presence of triphenylphosphine as the reductant. Systematic experiments and theoretical calculations revealed that the reaction proceeds via a tandem mechanism involving two Mo^VI^–Mo^IV^ catalytic cycles. In the first cycle, glycerol undergoes deoxydehydration to form allyl alcohol as a key intermediate. Successively, allyl alcohol undergoes C—O bond cleavage to generate allyl radicals, which underwent C(sp^3^)–C(sp^3^) homocoupling to yield 1,5‐hexadiene (Figure 3).

**FIGURE 4:**
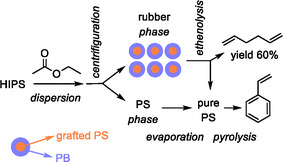
Schematic illustration of HIPS revalorization process [37].

A highly impactful perspective for accessing hydrocarbon *α*,*ω*‐dienes involves the chemical recycling of plastic waste, especially commodity polyolefins [26, 27, 28]. The selective chemical upcycling of plastic waste streams into valuable chemicals has the potential to play a key role in advancing a circular economy. Current valorization techniques for polyolefins involve pyrolysis [29], ethenolysis [30, 31], hydrogenolysis over metal catalysts [32, 33], and hydrocracking over bifunctional catalysts [34]. Unlike pyrolysis and mechanical recycling, ethenolysis (i.e., cross‐metathesis with ethylene) is potentially “green”, as it proceeds at low temperature and pressure with 100% atom economy [35]. The cross‐metathesis of internal olefins with ethylene was first termed “ethenolysis” by Bradshaw in 1967 [36]. This transformation has since been extensively investigated and it has been exploited commercially. Applications include the production of *ω*‐unsaturated carboxylic acids from fatty acids, as well as *α*,*ω*‐dienes from cyclic olefins and cross‐metathesis of ethylene and high‐molecular weight (MW) 1,4‐poly(1,3‐butadiene) (PB) rubber mediated by Ru catalysts [30]. More recently, in 2024, Marquez, De Vos et al. presented a strategy for the revalorization of high‐impact poly(styrene) (HIPS) to 1,5‐hexadiene as the major product [37]. HIPS is a two‐phase material consisting of a free PS matrix and rubber particles surrounded by covalently grafted PS. Among the variety of plastic waste generated, HIPS corresponds to 30%–40% of the worldwide PS production (7% of the global plastic market share) [38]. The authors first demonstrated a green fractionation process using ethyl acetate as an efficient solvent to separate the free PS from the PB rubber. The valorization of the rubber phase was then achieved using ethenolysis, where the grafted PS is separated from the rubber phase and further thermally degraded to pure styrene (selectivity of 70%), while the PB is split to yield 1,5‐hexadiene in the presence of ethylene and a Ru catalyst (4 h, 100°C, yield = 60%) (Figure 4).

**FIGURE 5:**
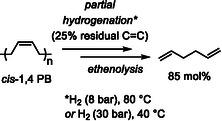
Consecutive hydrogenation–metathesis of PB rubber [39].

Precise control over the chain length of recovered *α*,*ω*‐dienes is a key challenge in the ethenolysis of PB rubber due to undesired isomerization occurring during the initial hydrogenation step. When PB is hydrogenated under low H_2_ pressure and high temperature, isomerization becomes significant, reaching up to 60 mol% of isomerization products. This process compromises product selectivity and leads to a broad distribution of dienes from C6 to C22. In 2025, De Vos et al. demonstrated that: (i) rational modification of consecutive hydrogenation metathesis conditions minimizes isomerization and steers selectivity toward the desired chain length of the *α*,*ω*‐diene products, yielding higher amount of C_4n+2_‐dienes (*n* = 1–5), and (ii) strict control over the degree of PB hydrogenation guides the product distribution [39]. For instance, for a partial hydrogenated PB containing 25% residual C=C bonds, successive ethenolysis gives a product distribution of 85 mol% 1,5‐hexadiene, 10 mol% C10 triene, and 5% C4 tetraene (Figure 5).

De Vos et al. employed olefin metathesis as a key step for degrading chlorinated polymers. Chlorinated polymer waste poses additional environmental challenges compared with non‐halogenated plastics. First, many additives historically associated with these materials are based on heavy metals or potentially endocrine‐disrupting compounds. Second, the high chlorine content (up to 57% of the total weight for PVC) makes end‐of‐life management particularly problematic. Consequently, there is a strong need for sustainable technologies capable of efficiently separating the halogen and carbon fractions while, whenever possible, valorizing the carbon feedstock. In this respect, De Vos and co‐workers have developed innovative strategies that are less energy‐demanding, avoid the generation of secondary waste streams and rely on catalysts free from heavy metals. Here, our intention is not to provide an exhaustive mechanistic discussion of these elegant approaches, but rather to highlight them as representative examples of promising emerging technologies. Readers interested in a more detailed understanding are strongly encouraged to access the original articles discussed below, together with the references therein.

**FIGURE 6:**
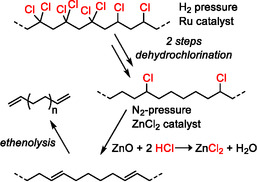
General reaction overview for conversion of highly chlorinated polymers into unsaturated PEs by dechlorination steps and successive ethenolysis to *α*,*ω*‐dienes [41].

In 2014, dehydrochlorination of chlorinated PE (CPE) (Cl = 36%) was first reported using tetrabutylphosphonium chloride [an ionic liquid known for catalyzing fast dehydrochlorination of cross‐linked poly(vinyl chloride) (PVC)]. This was followed by washing (neutralizing), drying, and ethenolysis using Hoveyda–Grubbs 2^nd^ generation catalyst, resulting in 34% conversion to *α*,*ω*‐dienes and *α*‐olefins. GC–MS analysis indicated that the reaction affords C6 to C20 hydrocarbons, including 1,6‐heptadiene (7.3%) and 1,5‐ hexadiene (6.6%) [40]. Later, the same authors extended this approach to other chlorinated polymers, including PVC and poly(vinylidene chloride) (PVDC), while the released HCl is simultaneously sequestered [41]. An entire range of chain lengths below C28 is produced from CPE, whereas longer carbon fragments are obtained via ethenolysis of PVC and PVDC–PVC copolymers. The process involves the conversion of highly chlorinated polymer into unsaturated polyolefins by sequential dechlorination steps, followed by ethenolysis to *α*,*ω*‐dienes. Careful pretreatment is required to prevent charring (i.e., formation of polyenes and polyaromatic species), while ZnO is employed as an HCl trap (Figure 6). It is worth noting that, mechanistically, the main challenge in converting chlorinated polyolefins into metathesis‐compatible substrates is that dehydrochlorination promotes the formation of conjugated polyenes, while the released HCl accelerates side reactions. Both HCl and residual chlorinated functionalities can negatively affect Ru catalysts. To address these issues, the process separates dechlorination and metathesis into separate steps. Tandem dehydrochlorination–hydrogenation limits the growth of extended polyene sequences and increases backbone saturation. By ensuing dehydrochlorination step in the absence of H_2_ and using ZnO as HCl scavenger to limit acid‐promoted side reactions, the intermediate products is transformed into fully dechlorinated polymers. Complete dechlorination is therefore achieved before ethenolysis, allowing metathesis to proceed on a chlorine‐free unsaturated polyolefin, while minimizing catalyst deactivation, char formation and the *α*,*ω*‐diene yield is maximized. Remarkably, the authors demonstrated the feasibility of the developed dechlorination sequence using a variety of PVC waste powders from the construction sector, including Pb‐stabilized PVC cable and Ca/Zn‐stabilized PVC pipe, and blister packs. Importantly, these waste materials contain substantial amounts of inorganic additives (i.e., CaCo_3_, Pb‐salt and Ca/Zn‐stearates), plasticizers (10%–20%), and other polymers such as PE and PP. Beneficially, after complete dehydrochlorination steps, full dechlorination was achieved for all waste materials. This results strongly support the potential practical applicability of this technology, demonstrating a remarkable tolerance toward the heterogeneous and chemically complex composition characteristic of real‐world PVC waste streams.

To summarize, Table 1 compiles the examples discussed in this section. The objective here is not to provide a critical or comparative analysis of the different approaches, nor to enter into a detailed mechanistic discussion involving thermodynamic constraints, rate‐determining steps, side‐product formation, or catalyst deactivation paths. This section instead highlights an emerging technological direction that may offer a long‐term perspective for non‐conjugated hydrocarbon dienes, stimulating further research and broadening their reactivity scope. It is evident that the synthesis of *α*,*ω*‐dienes from renewable resources and plastic upcycling is conceptually attractive and offers clear advantages in terms of environmental sustainability. However, its systemic advantage over conventional petrochemical routes have yet to be fully established, and future studies, supported by rigorous life‐cycle assessment and techno‐economic analysis – including, among other factors, carbon efficiency, energy demand, and catalyst footprint – will be crucial to quantitatively assess their environmental and economic viability. At the same time, rigorous and quantitative validation of the developed technologies on real‐world plastic waste streams remains essential before any realistic assessment of their practical industrial implementation can be made. Nonetheless, while these advances are still far from meeting the desirable industrial requirements for scalability, they provide a compelling *proof‐of‐principle* for alternative synthesis of hydrocarbon bifunctional synthons relevant to organic synthesis and polymer chemistry. A more critical and fully integrated perspective will, we hope, emerge in the near future.

**TABLE 1:** Schematic overview of chemical steps, catalysts, and yields for the production of *α*,*ω*‐dienes from biomass‐derived feedstock and via plastic upcycling.

Reactant	Practice/steps	Catalyst	Product	Yield, %	Remarks/operational conditions	Ref.
C6‐*α*,*ω*‐alkanediols	1) Esterification 2) Pyrolysis	Acetic acid	HXD	70.3	1) T up to 140°C 2) T = 450°C	[23]
C8‐*α*,*ω*‐alkanediols		Acetic acid	OTD	74.8		
C10‐*α*,*ω*‐alkanediols		Acetic acid	DCD	90.3		
C10‐*α*,*ω*‐alkanediols		Formic acid	DCD	8.9		
C10‐*α*,*ω*‐alkanediols		Propionic acid	DCD	78.9		
C10‐*α*,*ω*‐alkanediols		Butyric acid	DCD	67.3		
C10‐*α*,*ω*‐alkanediols		Isobutyric acid	DCD	71.5		
bio‐based ethanol	4 steps to styrene		HXD by‐product	na	HXD has the greatest impact on production costs	[24]
glycerol	1) Deoxydehydration 2) Deoxygenation 3) C(sp^3^)–C(sp^3^) homocoupling	Mo PPh_3_ as reductant	HXD	52	T = 220°C *t* = 30 min	[25]
HIPS	1) Fractionation 2) Ethenolysis	Ru	HXD	60	@ethenolysis T = 100°C *t* = 4 h	[37]
PB rubber	1) Hydrogenation 2) Ethenolysis	Ru	HXD	85	@partial hydrogenation step H_2_ = 8 bar, T = 80°C or H_2_ = 30 bar, T = 40°C	[39]
CPE	1) Dissolution/dehydrohalogenation 2) Ethenolysis	C_16_H_36_ClP Ru	HXD HPD	6.6 7.3		[40]
PVC	1) Dehydrochlorination 2) Hydrogenation 3) Ethenolysis	ZnCl_2_ Ru	Linear *α*,*ω*‐dienes		Metal oxides as a HCl trap	[41]
PVDC	1) Dehydrochlorination 2) Hydrogenation 3) Ethenolysis	ZnCl_2_ Ru	Linear *α*,*ω*‐dienes		Metal oxides as a HCl trap	[41]

Abbreviations: CPE, chlorinated PE; DCD, 1,9‐decadiene; HIPS, high‐impact poly(styrene), HPD, 1,6‐heptadiene, HXD, 1,5‐hexadiene; na, not available; PB, poly(1,3‐butadiene) rubber; PVC, poly(vinyl chloride); PVDC, poly(vinylidene chloride); OTD, 1,7‐octadiene.

### 2.3 Uses, Market Trend, and Limitations

Acyclic hydrocarbon *α*,*ω*‐dienes are valuable chemicals with potential applications across adhesives, lubricants, impact modifiers, pharmaceuticals and cross‐linking agents [42, 43, 44]. Owing to their dual reactivity and structural versatility, *α*,*ω*‐dienes also serve as substrates and (co)monomers in numerous fundamental chemical transformations. These include: (i) insertion (cyclo)polymerization [1], (ii) ADMET [2, 3], (iii) cyclization, cycloisomerization and cycloaddition [5], and (iv) hydroformylation [45].

Comprehensive and publicly accessible production volumes for non‐conjugated *α*,*ω*‐dienes are sparse, rendering a precise quantification of the global market elusive. While available data for specific molecules, such as 1,5‐hexadiene, offer useful industry insights, the limited information often presents conflicting reports on production scales and Compound Annual Growth Rate (CAGR), complicating a unified market assessment. CAGR projections for 2025–2032 fluctuate between 2.5% and 6.5%; while such a broad interval offers limited predictive precision, it confirms a sustained upward trajectory which reflects increasing demand across key applications in the automotive (seals, hoses), construction (roofing materials) and pharmaceutical industry [46, 47]. While current market data preclude granular forecasting, several significant obstacles constrain the utilization of non‐conjugated *α*,*ω*‐dienes. Foremost among these is the escalating regulatory scrutiny of *α*,*ω*‐dienes such as 1,5‐hexadiene, which is classified as a highly flammable and hazardous substance. Stringent updates to international safety standards have imposed stringent requirements for storage and transportation, thereby inflating operational costs. In addition, the production of 1,5‐hexadiene is still heavily dependent on petroleum‐derived feedstocks, exposing manufacturers to fluctuations in crude oil prices. These challenges have been further exacerbated by recent geopolitical instabilities and supply‐chain disruptions, which have intensified price fluctuations of key precursors such as 1,3‐butadiene. However, as discussed in the previous section, the prospect of accessing *α*,*ω*‐dienes through plastic waste upcycling or biomass‐derived feedstocks could strategically position these chemicals at the forefront of polymer science and green chemistry, addressing current limitations.

## 3 Insertion (cyclo)polymerization

The “*era of plastic*” began with the discovery of Ziegler–Natta catalysts and insertion olefin polymerization, which triggered major advances in polymer chemistry. Decades of research have led to an exceptionally detailed and refined understanding of insertion polymerization mechanisms. Advances in olefin polymerization have greatly expanded polymer (micro)structural diversity, and the design of materials that meet technological and sustainability needs [48, 49, 50, 51, 52]. Insertion olefin polymerization mediated by transition metal (pre)catalysts is by far the largest scale synthetic chemical transformation, accounting for most of the 400 million tons/year of plastics produced worldwide. Polyolefins, exemplified by HDPE, LDPE, LLDPE, and poly(propylene) (PP) account for more than 50% of global production capacity for all commodity plastics [53, 54, 55]. Polyolefins are versatile, strong, lightweight, and inexpensive, making them ubiquitous in modern life. While polyolefins are excellent materials, considerable effort focus on improving their properties through the incorporation of small amounts of unsaturation. Unsaturated moieties provide reactive sites along the main or side polymer chains that allow further functionalization with polar moieties in an otherwise nonpolar matrix and expanding their applicability in blending, adhesion, and dyeing. In addition, backbone unsaturation grants reactive sites for selective cleavage, thereby facilitating the development of circularity strategies for polyolefins. In fact, cleavable functionalities facilitate controlled deconstruction into value‐added fragments, which can be purified and repolymerized to fabricate recycled materials with properties comparable to the virgin ones [56, 57].

### 3.1 Mechanism Overview

**FIGURE 7:**
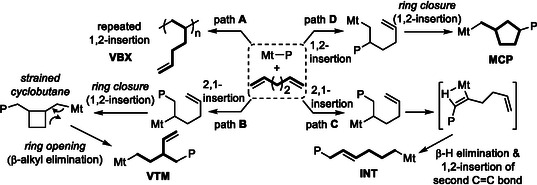
Known insertion modes of *α*,*ω*‐dienes: the example of 1,5‐hexadiene (Mt = transition metal, P = growing polymer chain).

The insertion polymerization of *α*,*ω*‐dienes mediated by transition metal catalysts is a convenient and straightforward way to synthesize unsaturated polyolefins with internal and/or pendant C=C double bond. Indeed, 1,2‐ (or 2,1‐) insertion of an *α*,*ω*‐diene installs an alkyl branch with a terminal C=C bond in the repeating unit (Figure 7 ‐ path A, hereafter referred to VBX for 1,5‐hexadiene and VHX for 1,7‐octadiene). Pendant vinyl groups may also be formed through a 2,1‐insertion and subsequent 1,2‐insertion of the remaining C=C bond (*ring closure*) to afford, in the case of 1,5‐hexadiene, a strained cyclobutane ring, followed by *β*‐alkyl activation (*ring opening* – Figure 7 ‐ path B) [58]. The result of this mechanism is the placement of a 3‐vinyl‐tetramethylene unit (hereafter referred to VTM). Further, the insertion of an *α*,*ω*‐diene can proceed via a double linear insertion mechanism, which results in linear repeating units with internal C=C bonds (hereafter referred to INT – Figure 7 ‐ path C). This uncommon insertion mode was first reported by Hasegawa et al. [59], and only recently revisited and further validated by Huang [60], and Leone [61]. Huang and coworkers demonstrated that the linear diene enchainment involves 2,1‐insertion of the first vinyl group into a metal–carbon bond and *β*‐H elimination, followed by 1,2‐insertion of the second vinyl group into a carbon–hydrogen bond [60].

**FIGURE 8:**
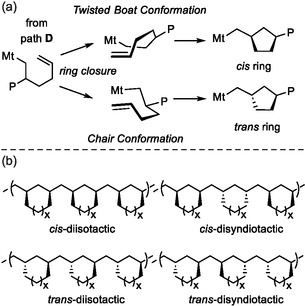
(a) Diastereoselectivity in cyclopolymerization of 1,5‐hexadiene and (b) Cyclopolymers structures of maximum order (*x* = 0, 1, or 2).

Moreover, it is well established that the insertion polymerization of *α*,*ω*‐dienes also yields saturated polymers with cycloalkane enchained units. The ring enchainement is due to a favorable 1,2‐insertion and subsequent cyclization through 1,2‐insertion of the second C=C bond [that is, methylene‐1,3‐cyclopentane, hereafter referred to MCP for 1,5‐hexadiene – Figure 7 ‐ path D]. The diastereoselectivity of the cyclization step, governed by conformational constraints of the transition state, dictates the configuration (*cis* or *trans*) of repeating cyclic units [62]. Insertion of the first and second double bonds via the same enantioface yields the *cis*‐fused ring, whereas coordination and insertion of opposite enantiofaces yields the *trans* isomer (Figure 8a). At least, therefore, cyclopolymers may exhibit four microstructures, that is, *cis* or *trans* diisotactic and *cis* or *trans* disyndiotactic (Figure 8b) [63]. A comprehensive review by Pasini and Takeuchi provides a detailed account of *α*,*ω*‐diene cyclopolymerization [1]; accordingly, these examples are discussed briefly here. The following subsections survey recent chemistries, transition metal catalysts, and (co)polymers, with particular emphasis on how their microstructures and properties can be exploited for real‐world applications. All (pre)catalysts are classified by transition metal group. Unless otherwise stated, the structures illustrated correspond to precatalysts, which require activation – typically with methylaluminoxane (MAO), an aluminum alkyl, or a borate – to form the catalytically active species.

### 3.2 Optically Active Polymers

The synthesis of optically active (chiral) polyolefins dates back to the 1950s [64, 65, 66], and it is a fascinating yet relatively underexplored area of olefin chemistry [67]. Chiral polyolefins fall into two major categories: those in which chirality arises from polymer tacticity, and those in which it is introduced by incorporating chiral units into the monomer structure [68, 69]. Applications of chiral polyolefins range from chiral recognition materials used as stationary phases in high‐performance liquid chromatography for separating racemic mixtures to biomimetic macromolecules that mimic peptides and DNA [70].

**FIGURE 9:**
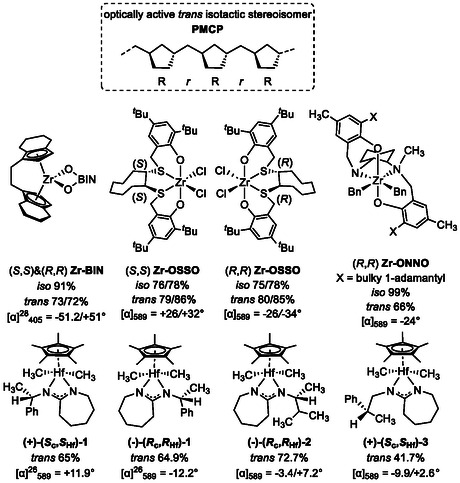
The optically active *trans* isotactic **PMCP** together with selected Group IV (pre)catalysts.

Among the four maximally cyclic structures obtainable from *α*,*ω*‐dienes (Figure 8b), the *trans* diisotactic configuration of poly(methylene‐1,3‐cyclopentane) (**PMCP**) is chiral due to its main‐chain stereochemistry – namely, the predominance of *trans* rings and the high degree of isotacticity, that is the same relative configuration at every other stereocenter. While the cyclopolymerization of 1,5‐hexadiene was first reported by Marvel [71, 72], and Makowski at the turn of the 1960s [73], only in the early 1990s Coates and Waymouth reported the enantioselective cyclopolymerization of 1,5‐hexadiene. This was mediated by the chiral precatalyst (–)‐(*R*)‐ethylenebis(tetra‐hydroindenyl)zirconium (*R*)‐binaphtholate (**Zr‐BIN** in Figure 9) to yield the optically active **PMCP** with almost 70% *trans* rings (Figure 9) [74, 75]. Successively, a variety of chiral Group IV metal catalysts have been successfully synthesized. Notable examples include Zr complexes bearing [ONNO] (*R*,*R*)‐salan ligands (**Zr‐ONNO**) [76], and [OSSO]‐type ligand (**Zr‐OSSO)** (Figure 9) [77].

**FIGURE 10:**
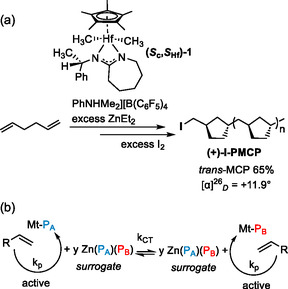
(a) LCCTP of 1,5‐hexadiene. Synthesis of **PMCP** and **I‐PMCP** optically active materials and (b) mechanism of LCCTP. (P_A_ = P_B_ = polymeryl chain. Mt = transition metal propagator).

Recently, major advances have been reported by Sita and coworkers. In 2020, they described the enantioselective living coordinative chain‐transfer polymerization (LCCTP) of 1,5‐hexadiene using a homochiral *C*_1_–cyclopentadienyl caproamidinate (CPAM) Hf precatalyst, readily accessible in either enantiomeric (*S*_C_,*S*_Hf_) and (*R*_C_,*R*_Hf_) form [78]. In combination with [PhNHMe_2_][B(C_6_F_5_)_4_] as co‐initiator and ZnEt_2_ as chain‐transfer agent (CTA), the **(*S***_**C**_ **,*S***_**Hf**_ **)‐1** precatalyst gives the isotactic *cis*,*trans*‐**PMCP** with only a small fraction of pendant side chains (less than 2%) and up to 65% *trans* cyclic units (Figure 10a). Significant optical activity is observed, with a specific rotation of [*α*]^26^_D_ = +11.9° (*c* = 2, CHCl_3_), which decreases to +7.5° as the content of enchained *trans* units drops to 57.3%. Iodo‐terminated isotactic *cis*/*trans* **I‐PMCPs** were successfully synthesized by quenching the polymerization with excess molecular iodine. This results arises from the distinctive mechanism of LCCTP, that is, “one‐chain‐per‐active‐site”, in which rapid and reversible polymeryl chain transfer occurs between a small population of active transition‐metal propagation species and a much larger pool of dormant main‐group‐metal “surrogate” chain‐growth centers (Figure 10b) [79]. This dynamic exchange ensures controlled chain‐growth and facilitates the incorporation of target end groups. End‐functionalized polymers have attracted increasing interest for the development of nanostructured and advanced functional materials [80, 81].

In 2024, Sita et al. reported the synthesis of two novel homochiral and diastereomerically pure Hf complexes (i.e., **(*R***_**C**_ **,*R***_**Hf**_ **)−2 (*S***_**C**_ **,*S***_**Hf**_ **)‐3** in Figure 9) where the bicyclic CPAM coordination ensures exceptional configurational stability at the metal center [82]. Differences in the ligand substituents modulate the polymer stereochemical microstructure; **(*R***_**C**_ **,*R***_**Hf**_ **)‐2** yields a polymer with the highest content of *trans*‐MCP units (up to 72.7%) and maximum chirality and optical activity.

**FIGURE 11:**
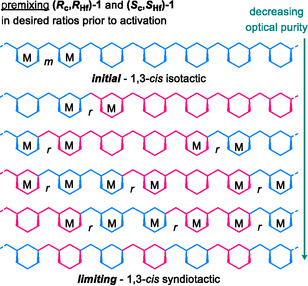
Optical purity as a programmable variable. Meso (*m*) or racemic (*r*) labels to keep track of the relationship between adjacent stereocenters on two different rings, while M and R correspond to the respective *cis* and *trans* stereochemical relationships of adjacent stereocenters within each ring. Adapted from ref. [83].

**FIGURE 12:**
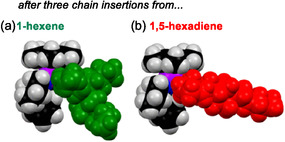
Space‐filled representations of computationally derived energy‐minimized structures for cationic propagating species derived from **(*S***_**C**_ **,*S***_**Hf**_ **)‐1** and after three chain insertions of (a) 1‐hexene and (b) 1,5‐hexadiene. Green and red denote space‐filling of the nascent isotactic poly(1‐hexene) and isotactic *cis*/*trans* PMCP chains, respectively. Adapted from ref. [84] (License Number 6 267 560 085 248).

In related studies, Sita and coworkers provided fundamental insights into stereocontrolled LCCTP and living coordinative polymerization (LCP) of *α*,*ω*‐dienes and analog *α*‐olefins. A strategy of using optical purity (i.e., enantiomeric composition) of the chiral *C*_1_–symmetric Hf precatalyst was validated as a programmable parameter for controlling polyolefin tacticity. This approach revealed a correlation between the diastereoselectivity and efficiency of the cyclization step and the extent of site epimerization, that is, reversible change in configuration of the active Hf species, which can erode polymerization stereocontrol. Different stereochemical grades of *cis*‐poly(methylene‐1,3‐cyclohexane) (***cis*‐PMCH**), spanning isotactic to syndiotactic forms, were synthesized by premixing the pure homochiral **(*R***_**C**_ **,*R***_**Hf**_ **)‐1** and **(*S***_**C**_ **,*S***_**Hf**_ **)‐1** enantiomers in different ratios prior to activation (Figure 11) [83]. Remarkably, all attempts to carry out the enantioselective LCCTP of 1‐hexene using the same Hf precatalyst and conditions successfully used for 1,5‐hexadiene and 1,6‐heptadiene failed. The authors attributed this unprecedented difference in polymerizability between the two monomers (*α*‐olefin *vs α*,*ω*‐diene) to the distinct conformational freedom of the growing polyolefin chains (Figure 12). In **PMCP** formation, the rigid growing chain (red ‐ Figure 12) facilitates reversible chain transfer, whereas the unrestricted conformational flexibility of the C4 side chains in poly(1‐hexene) (green ‐ Figure 12) generates a steric barrier that prohibits chain transfer after just a few monomer insertions [84].

### 3.3 Copolymerization of α,ω‐Dienes Using Group IV (pre)Catalysts

While cyclopolymers rich in isotactic *trans* MCP units are particularly intriguing due to their optical activity, linear polymers with unsaturated pendant side chains are desirable to meet specific properties and applications. Key properties of interest include crystallinity, transparency, processability, flexibility, tensile strength, melting point (*T*_m_), and glass transition temperature (*T*_g_). One effective option is the copolymerization of two or more monomers to access polymeric materials exhibiting properties intermediate between those of distinct homopolymers. However, introducing multiple monomers to the reaction feed mixture increases the complexity of reaction kinetics, as relative propagation rates strongly depend on the monomer structure. Copolymerization of *α*,*ω*‐dienes with ethylene, *α*‐olefins or 1,3‐dienes is particularly challenging due to their unmatched reactivities, frequently resulting in a mixture of homopolymers [85], compositional heterogeneity (*composition drift*) or low diene incorporation [86, 87]. In addition, under diene concentrated conditions, gel formation occurs due to severe side intramolecular cross‐linking. This, in turn, causes both initiation and propagation to become diffusion‐controlled at high (co)monomer conversion. Compositional heterogeneity and cross‐linking are often detrimental to optical and rheological properties, but they can nevertheless be strategically exploited to enhance polymer performance and expand their application scope [88]. One example is cable insulation, where they are considered potential alternatives to conventional cross‐linked PE (XLPE) produced via peroxide‐ or radiation‐initiated cross‐linking [89].

The copolymerization of *α*,*ω*‐dienes with ethylene is one of the most investigated reactions, as the installation of unsaturated pendant branches or cyclic units disrupts chain packing and reduces PE crystallinity. For example, Imanishi et al. found that the MCP unit is incorporated into the crystalline phase of PE by partially changing the *trans* zigzag chain into a gauche conformation, inducing a transformation of the orthorhombic crystal into a pseudohexagonal crystal [90, 91]. Moreover, alkyl chain branches improved PE flexibility, processability, and impact resistance, while also providing reactive sites for further chemical functionalization.

#### 3.3.1 Heterogeneous Catalysts

Heterogeneous TiCl_4_/MgCl_2_ catalysts are the industrial benchmark for PP production due to their exceptional activity, stability, and control over polymer morphology. These features ensure their continued dominance in commodity polyolefin manufacturing [92]. Heterogeneous catalysts are typically favored over molecular catalysts in industrial slurry polymerizations because they prevent the formation of fine polymer particles and reactor fouling. Nevertheless, examples on the topic covered in this review are scarce. TiCl_4_/MgCl_2_ has been recently used as catalyst for the copolymerization of ethylene with 1,5‐hexadiene by Rahmatiyan et al. [93], and for the copolymerization of ethylene (or propylene) with 1,9‐decadiene by Dong et al. [94, 95, 96]. Semicrystalline, branched copolymers with low diene incorporation (<2 mol%) are typically obtained. Notably, the resulting long chain branched PPs exhibit enhanced low‐frequency elasticity (i.e., a greater elastic response under slow deformation), pronounced shear‐thinning (i.e., a decrease in viscosity under increasing shear rate), and improved melt strength compared to linear PP [94]. This rheological behavior is particularly advantageous for blow molding and film extrusion to manufacture lightweight packaging subjected to static or quasi‐static loads.

Recently, Yao et al. explored the reactivity of heterogeneous TiCl_4_ catalysts in the copolymerization of 1,5‐hexadiene with *α*‐olefins (i.e., 1‐hexene and 1‐dodecene). They obtained cross‐linked polyolefins, likely due to favorable intramolecular side reactions, which exhibit mechanical strength of 0.19 MPa, fracture toughness up to 130 MJ m^−3^, and robust shear stability. The entanglement network leads to a drag reduction rate, defined as the percentage decrease in flow resistance after shear exposure. As a result, these polymers are promising additives for reducing turbulence and friction in fluid flow [97]. Similarly, using the Mg(OEt)_2_/FeCl_3_/TiCl_4_/DNBP (DNBP = di‐*n*‐butyl‐phthalate) heterogeneous catalyst, Mirmohammadi et al. synthesized a 1‐hexene/1,5‐hexadiene copolymer with 24 mol% unsaturated units and 5 mol% MCP units. This material proved highly effective as an impact modifier for PS, dramatically increasing its impact strength to up to 140 J m^−1^, more than an order of magnitude higher than that of neat PS (8 J m^−1^) [42].

#### 3.3.2 Molecular Catalysts

Packaging is the most critical application of ethylene (co)polymer films. Strain‐hardening (i.e., increase in the material's resistance to deformation as it is stretched) and shear‐thinning are essential to fabricate stable blown films. Current technologies introduce long chain branching (LCB) into the PE backbone to meet extensional and shear rheology requirements. LCBs significantly alter the melt flow by promoting entanglements between polymer chains. This results in shear‐thinning and increased melt strength as branches prevent the polymer from sagging or tearing during high‐stress blown film extrusion or thermoforming processes. The copolymerization of ethylene with *α*,*ω*‐dienes mediated by molecular Group IV complexes is one of the most promising strategies for introducing LCBs. Particularly, the dimethyl(pyridylamido) Hf precatalyst (hereafter named **Hf1**), originally reported by Dow and Symyx for the synthesis of PP and olefin block copolymer [98], have been largely used due to its excellent catalytic activity, effective stereocontrol and ability to insert long chain *α*‐olefins and non‐conjugated dienes. While studies up to 2018 have been comprehensively reviewed by Pasini and Takeuchi [1], this section covers advances reported thereafter.

**FIGURE 13:**
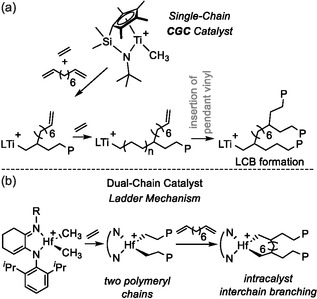
1,9‐Decadiene reactions with (a) the single‐chain constrained (**CGC**) and (b) the dual‐chain imino‐enamido Hf precatalyst [100].

In 2026, Qiu et al. demonstrated that trace amount of 1,9‐decadiene into the copolymerization of ethylene with 1‐octene installs LCBs which significantly increase the copolymer's zero‐shear viscosity, enhances shear‐thinning, and elevates low‐frequency storage modulus [99]. Similarly, Froese et al. installed LCBs using 1,9‐decadiene as comonomer and a dual‐chain catalyst [100], that is a metal cation capable of propagating two polymer chains simultaneously [101]. Indeed, while a single‐chain catalyst inserts only one vinyl group of the *α*,*ω*‐diene, leaving the other as a pendant group (Figure 13a), a dual‐chain catalyst reacts with one end of the diene but can then either (i) insert the comonomer that moves the vinyl‐terminated branch away from the metal center (*traditional insertion mechanism*) or (ii) engage the second vinyl group across the second growing chain, forming a ladder structure (*ladder mechanism –* Figure 13b). Specifically, the authors investigated the semibatch copolymerization of ethylene with 1,9‐decadiene (less than 1 mol%) using an imino‐enamido Hf precatalyst at 150°C. NMR spectroscopy, kinetics studies and models provided strong evidence for C6 branching: the diene couples two polymer chains through the *ladder mechanism*. The ladder polymer with 0.07 LCB per 1000 C, corresponding to roughly one LCB every seven polymer chains, exhibits strong resistance to flow under slow deformation and pronounced shear‐thinning, which increase with diene content and exceed those of LDPE grades. As noted above, such rheological properties are particularly advantageous for maintaining dimensional stability during post‐processing cooling.

**FIGURE 14:**
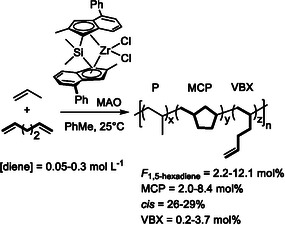
Copolymerization of propylene with 1,5‐hexadiene catalyzed by *ansa*‐zirconocene precatalyst (*F*_1,5‐hexadiene_ is the total incorporation data) [102].

*α*,*ω*‐Dienes have also emerged as highly effective comonomers for the synthesis of isotactic random and block PP (co)polymers. The copolymerization of 1,5‐hexadiene with propylene using *ansa*‐zirconocene precatalyst has been recently investigated (Figure 14) [102]. The obtained high–MW, isotactic copolymers feature a random distribution of MCP units (2.0–8.4 mol%), over 70% of which adopt a *trans* configuration, along with unsaturated butylene side groups (VBX = 0.2–3.7 mol%). The incorporation of both cyclic and pendant units in the *i*PP significantly affects the copolymer's *T*_m_, *T*_g_ and crystallinity. High concentrations of enchained diene units, which are excluded from *i*PP crystals, shorten crystallizable segments and reduced overall crystallinity. The copolymers with the highest diene content show a granular morphology in AFM images, with small crystallites dispersed in an amorphous matrix that act as physical cross‐links and ensure elastic recovery as high as 70%–90%, at least at low deformation.

**FIGURE 15:**
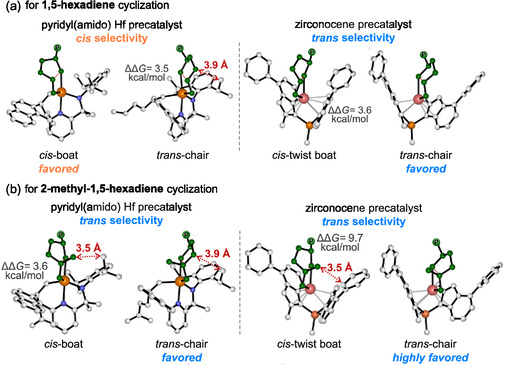
(a) DFT optimized geometries of the favored transition state (TS) for *cis*‐ and *trans*‐cyclization of 1,5‐hexadiene; (b) DFT optimized geometries of the favored TS for *cis*‐ and *trans*‐cyclization of 2‐methyl‐1,5‐hexadiene. Adapted from ref. [103]. (License Number 6 267 560 641 392).

Talarico et al. more recently conducted a combined experimental and theoretical study on the copolymerization of propylene with either 1,5‐hexadiene or 2‐methyl‐1,5‐hexadiene using *C*_2_‐, *C*_s_‐metallocene and pyridyl(amido) Hf catalysts [103]. For *ansa*‐metallocenes, which typically favor *trans*‐cyclization of 1,5‐hexadiene [1], a methyl group at the 2‐position further enhances *trans*‐selectivity, likely due to steric repulsion with the ligand. In contrast, pyridyl(amido) Hf catalysts, previously reported to favor *cis*‐cyclization [1], exhibited reversed diastereoselectivity with 2‐methyl‐1,5‐hexadiene as it preferentially enchained *trans* units (Figure 15).

**FIGURE 16:**
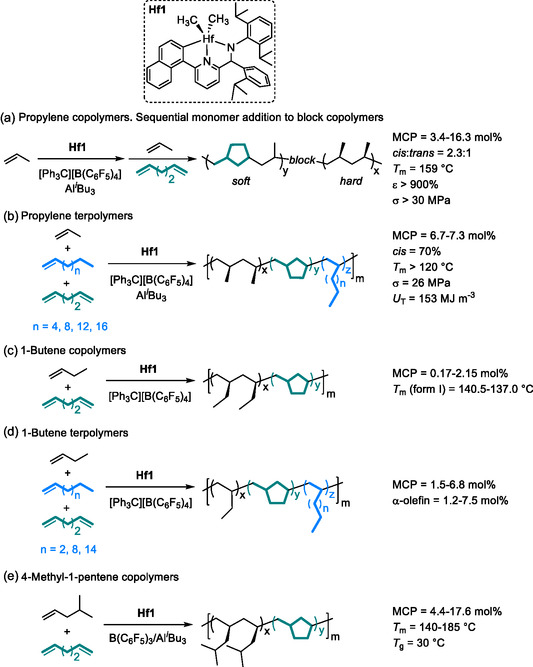
Dimethyl(pyridylamido) **Hf1** precatalyst and (block) copolymers from 1,5‐hexadiene and *α*‐olefins. (a) Propylene block copolymers via sequential monomer additions; (b) Propylene terpolymers; (c) 1‐Butene copolymers; (d) 1‐Butene terpolymers; (e) 4‐Methyl‐1‐pentene copolymers.

In 2024, Pan, Ma et al. synthesized diblock copolymers using **Hf1**, in combination with [Ph_3_C][B(C_6_F_5_)_4_] as the cocatalyst and Al^*i*^Bu_3_ as CTA, via coordinative chain‐transfer polymerization (CCTP) and sequential monomer additions (Figure 16a). The synthesized block copolymers consist of a *hard i*PP first block and a second *soft* block of random poly(propylene‐*co*‐MCP) segments, in which the diene (from 3.4 to 16.3 mol%) forms exclusively rigid cyclic MCP units (*cis*:*trans* = 2.3:1). The block copolymers exhibited *T*_m_s over 150°C, close to that of pure *i*PP, high yield stress (>30 MPa) and elongation at break > 900%, significantly exceeding that of *i*PP [104].

**Hf1** has also been utilized as precatalyst for terpolymerization of 1,5‐hexadiene with propylene and *α*‐olefins (from C8 to C20) (Figure 16b) [105]. In the resultant polymers, the *α*,*ω*‐diene installs rigid cyclic units (MCP = 6.7–7.3 mol%, *cis* selectivity = 70%), whereas the *α*‐olefin introduces long, flexible pendant branches (*α*‐olefin = 11.4–15.5 mol%). This complementary structural combination enables excellent elastic recovery (up to 94% even at strain as high as 1000%), high tensile strength (up to 26 MPa), elongation at break up to 1360%, pronounced strain‐hardening near fracture, and outstanding toughness (*U*_T_ = 153 MJ m^−3^). Moreover, the materials show *T*_m_s over 120°C, significantly exceeding those of commercial ethylene/1‐octene plastomers Queo 8201 produced by Borealis (*T*_m_ = 73°C), while maintaining comparable tensile strength. Overall, these polymers behave as robust thermoplastic elastomers; they combine high toughness and excellent flexibility, thereby resulting in improved resistance to mechanical damage. As such, they are promising candidates for advanced engineering applications, including soft robotics, damping systems, stretchable sensors, and wearable electronics [48].

In contrast, the copolymerization of *α*,*ω*‐dienes with 1‐butene is largely underexplored, which is somewhat surprising given the inherently attractive properties of poly(1‐butene); indeed, it combines high toughness, tear strength, and thermal endurance with outstanding creep and impact resistance. As such, poly(1‐butene) is particularly suitable for demanding applications such as high‐pressure tanks, pumps, and hot‐water piping systems [106]. In addition, poly(1‐butene) is a polymorphic semicrystalline polymer capable of adopting various helical conformations and crystallizing into different unit cell structures, depending on the solidification conditions. It can generate diverse crystallite structures, including twined hexagonal form I, untwined hexagonal form I′, tetragonal form II, and orthorhombic form III, which strongly dictates polymer properties and applications. In this respect, the copolymerization of 1‐butene is a key strategy to adjust the crystallite formed and tailor material performance [107, 108, 109]. 1‐Butene copolymers with 1,5‐hexadiene were first synthesized and characterized by Li, Ma et al. using **Hf1/**[Ph_3_C](B(C_6_F_5_)_4_] (Figure 16c) [110]. The copolymerization proceeds via quantitative diene cyclization, thus forming random poly(1‐butene‐*ran*‐MCP)s (MCP from 0.17 to 2.15 mol%). The incorporation of cyclic units disturbs the 1‐butene chain regularity and significantly dictates the crystallization kinetics. WAXD measurements reveal that the polymorphism of crystallization from the melt greatly depends on the concentration of MCP units and crystallization temperature. MCP units accelerates the II→I phase transition; the packing constraints and altered chain mobility imposed by the cyclic units modulate nucleation and crystal growth, affect crystallization polymorphism under both quiescent and flow conditions, and introduce a memory effect in the copolymers, even above the equilibrium melting temperature [111, 112]. Later, it is proved that the onset of plastic deformation triggers the II→I phase transition and that the transformed form I significantly increases the strain‐hardening modulus by two orders of magnitude compared to form II. This indicates that form I crystallites act as physical knots or cross‐linking within the molecular network [113].

Terpolymerization of 1,5‐hexadiene, 1‐butene and *α*‐olefins (from C6 to C18) has been further demonstrated a viable strategy to balance strength and elasticity in 1‐butene elastomers and to elucidate the role of polymorphism in physical cross‐links (Figure 16d) [114]. 1,5‐Hexadiene is predominantly incorporated as MCP repeating units (from 1.5 to 6.8 mol%), whereas the *α*‐olefin content (from 1.2 to 7.5 mol%) depends strongly on chain length and governs crystallization behavior and polymorphism. The terpolymers span from tough thermoplastics to highly ductile elastomers, depending on the relative prevalence of trigonal or tetragonal crystallites. Trigonal crystallites act as efficient, deformation‐resistant physical cross‐links, anchoring the amorphous segments during deformation while simultaneously enhancing elastic recovery, tensile strength and toughness. In contrast, tetragonal crystallites, due to their higher chain mobility, are inherently unstable and more prone to slippage during stretching than trigonal crystals, thereby limiting recovery upon unloading.

**Hf1**, activated with B(C_6_F_5_)_3_, is also effective for the cyclocopolymerization of 1,5‐hexadiene with 4‐methyl‐1‐pentene (Figure 16e). The resulting copolymers are composed of MCP units (from 4.4 to 17.6 mol%) and long isotactic 4‐methyl‐1‐pentene repeating units. The copolymers exhibit *T*_g_s close to 30°C and a glassy‐ductile behavior. Interestingly, the copolymers with cyclic repeating units below 12 mol% crystallize from the polymerization solution in the form of isotactic poly(4‐methyl‐1‐pentene) with chains in 4/1 helical conformation (typically referred to form II), and from the melt in the stable form with chains in 7/2 helical conformation (typically referred to form I). In contrast, the copolymer with 17.6 mol% of MCP units is amorphous; it does not crystallize from the polymerization solution or by cooling the melt from high temperature [115].

**FIGURE 17:**
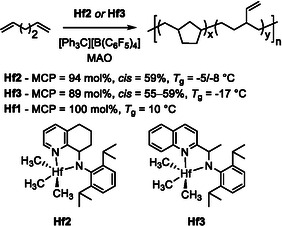
Polymerization of 1,5‐hexadiene catalyzed by amido‐quinoline and amido‐trihydroquinoline **Hf2** and **Hf3** (pre)catalysts [116].

In contrast, the homopolymerization of 1,5‐hexadiene using amido‐quinoline or amido‐trihydroquinoline Hf precatalysts (**Hf2** and **Hf3**, respectively – Figure 17) affords polymers composed of cyclic and uncyclized (predominantly of VTM‐type – Figure 7) random repeating units in a 9:1 ratio [116]. No pendant 3‐butenyl VBX units were detected, indicating that consecutive 1,2‐insertion of 1,5‐hexadiene does not occur (Figure 7 – path A). The content of *cis*‐fused MCP units is from 55.4 to 59.1 mol%, consistent with a preference for the twisted boat conformation (Figure 8a). The polymers are soluble, gel‐free with *T*_g_s from −5°C to −17°C. The presence of unsaturated VTM units was further explored for post‐polymerization modification (*vide infra*).

**FIGURE 18:**
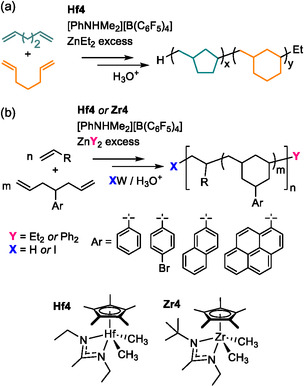
(a) Synthesis of poly(methylene‐1,3‐cyclopentane‐*ran*‐methylene‐1,3‐cyclohexane)s via LCCTP and (b) synthetic paths to multivariate, end‐group and main‐chain functionalized polyolefins via LCCTT of *α*‐olefins (R = CH_3_, C_4_H_9_, CH_2_CH(CH_3_)CH_2_CH_3_) and 4‐aryl‐1,6‐heptadienes [118].

A further class of Hf initiator comprises cyclopentadienyl–amidinate half‐sandwich complexes, previously highlighted for their effectiveness in producing optically active polymers from 1,5‐hexadiene. The (η^5^‐C_5_Me_5_)[(N,N)‐κ^2^‐(NEt)_2_C(Me)]Hf(Me)_2_ complex (**Hf4** – Figure 18a) in combination with [PhNHMe_2_][B(C_6_F_5_)_4_] as activator (1 equiv. to Hf) and ZnEt_2_ as a chain transfer agent (CTA, 5 or 20 equiv. to Hf) was also investigated for the cyclocopolymerization of 1,5‐hexadiene and 1,6‐heptadiene. Atactic *cis,trans*‐poly(methylene‐1,3‐cyclopentane‐ran‐methylene‐1,3‐cyclohexane)s with tunable ratios of 5‐ and 6‐membered units were successfully synthesized by changing the comonomer feed ratio. Unlike atactic cis,trans‐PMCP, these copolymers are fully amorphous, with *T*_g_s from 6.8°C to 87.9°C increasing with cyclohexane content. Even low levels of 6‐membered rings disrupt **PMCP** crystallinity and markedly increase chain stiffness relative to the conformational flexibility of the all‐5‐membered ring chain of **PMCP** [117]. **Hf4** as well as its Zr analog (for simplicity hereafter named **Zr4** –Figure 18b) was also utilized to copolymerize 4‐aryl‐1,6‐heptadienes, readily obtained from aryl carboxaldehydes, with 1‐alkenes (i.e., propylene, 1‐hexene and 4‐methyl‐1‐pentene) [118]. Copolymerization of 4‐aryl‐1,6‐heptadienes with 1‐alkenes gives access to statistical copolymers, end‐functionalized polymers (using excess ZnEt_2_ or ZnPh_2_ as CTA), or telechelic polymers upon quenching with I_2_ or simple protic workup. The obtained polymers, semi‐crystalline or amorphous with *T*_g_ values from −61°C to 45°C, are early examples of polyolefins with both telechelic and main‐chain functionalities with orthogonal reactivity for post‐functionalization.

**FIGURE 19:**
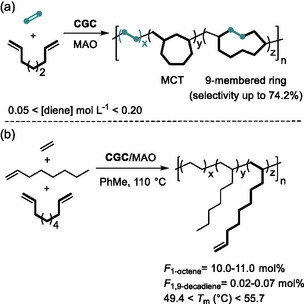
(a) Copolymerization of 1,7‐octadiene with ethylene catalyzed [123], and (b) terpolymerization of 1,9‐decadiene with ethylene and 1‐octene catalyzed by **CGC** [120].

Beyond Hf and Zr precatalysts, the dimethysilylene(tetramethylcyclopentadienyl)(*N*‐*tert*‐butyl)titanium dichloride catalyst (the constrained geometry catalyst ‐ **CGC**) is well known for its high activity and ability to yield LLDPE and other specialty polyolefins with excellent control over branching and molecular architecture. Across the timeframe of this review, **CGC** has been extensively used for insertion copolymerization of C8 and C10 *α*,*ω*‐dienes [119, 120, 121, 122]. Yet in 2004, Naga and Toyota discovered a unique insertion mode of 1,7‐octadiene using **CGC**. They found that the copolymerization of 1,7‐octadiene with ethylene mediated by **CGC** yields copolymers in which the diene (up to 30.6 mol%) is predominantly enchained as methylene‐1,3‐cyclononylene (9‐membered ring) repeating units, in addition to minor methylene‐1,3‐cycloheptylene (7‐membered ring ‐ MCT) structure and uncyclized 1,2‐inserted units (not shown in Figure 19a) [123]. The formation of the unique 9‐membered rings is ascribed to the cyclization of the penultimate 1,2‐inserted diene unit after a single ethylene insertion. In 2018, Soares et al. further investigated the copolymerization of 1,7‐octadiene with ethylene using **CGC** at 110°C. They obtained highly crystalline copolymers (detailed microstructure data are not available) with *T*_m_s and tensile properties close to those of HDPE, i.e, *T*_m_s from 129 to 132°C, tensile strength up to 30.7 MPa, Young's modulus > 790 MPa, and elongation at break exceeding 500%. Interestingly, the copolymers exhibited a higher dielectric constant than parent PE across all measured frequencies. This enhancement is attributed to increased amorphous content and reduced viscosity, which enlarge the free volume and facilitate dipole alignment under an applied electric field. Accordingly, these copolymers exhibit enhanced dielectric performance and are promising candidates for electronic packaging applications, where improved conductivity and dielectric properties help mitigate static charge accumulation [119].

In 2020, Liu, Wang et al. investigated the terpolymerization of 1,9‐decadiene with ethylene and 1‐octene using **CGC** (Figure 19b) [120]. The terpolymerization proceeds without diene cyclization and affords polymers with unsaturated pendant chains, which were then mixed with a peroxide curing agent to fabricate cross‐linked polymers. The cured polymers exhibit high transmittance (91% at 380–1100 nm), good mechanical and adhesive strength (fracture strength = 6.6 MPa, elongation at break up to 725%, peel strength of 121 N cm^−1^), water‐vapor permeability, and high thermal stability (initial decomposition temperature at 415°C), comparable or even superior to the commercial encapsulant ethylene–vinyl acetate (EVA). Moreover, the light transmittance does not change after UV irradiation at 120 kW h m^−2^ and aging at 85°C and is still at 87% after a highly accelerated stress test at 121°C and relative humidity of 100%. All in all, these cross‐linked polymers have excellent prospects for use as an encapsulation material for solar cell lamination. They also provide strong adhesion and effective long‐term protection against moisture, which are critical for module durability.

**FIGURE 20:**
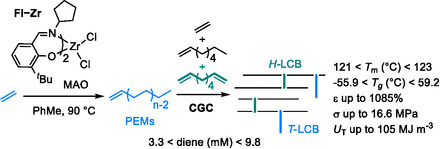
Tandem catalytic synthesis of dual long‐chain branched polyolefin elastomers from ethylene, 1‐octene, and 1,7‐octadiene [121].

Comb, cross‐linked elastomers have been also synthesized using **CGC** through the tetrapolymerization of ethylene, 1‐octene, 1,7‐octadiene, and PE macromonomers (PEMs) [121]. The synthetic strategy first involves the polymerization of ethylene using (3‐^*t*^Bu‐2–OC_6_H_3_CH = NC_5_H_9_)_2_ZrCl_2_ (**FI‐Zr**) as a catalyst to produce PEMs (*M*_w_ = 5.7 kDa) (Figure 20). PEMs were subsequently tetrapolymerized with ethylene, 1‐octene, and 1,7‐octadiene using **CGC** to afford branched polyolefin elastomers featuring both crystalline long methylene branches (referred to *T*‐LCBs, 0.5–0.6 × chain), which act as physical knots, and covalent chain bridges due to intermolecular cross‐linking of the last inserted 1,7‐octadiene unit (referred to *H*‐LCBs, 0.5–1.6 × chain). The obtained polymers have *T*_m_s between 120°C and 125°C, tensile strength (σ) up to 16.6 MPa, elongation at break (*ε*) of 1000%, toughness (*U*_T_) up to 105 MJ m^−3^ and elastic recovery as high as 73.7%. Notably, the polymers also exhibit great reprocessability, retaining up to 96% of their original tensile strength and ultimate elongation even after five hot‐pressing cycles.

**FIGURE 21:**
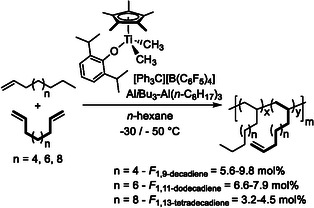
Copolymerization of C10, C12 and C14 *α*,*ω*‐dienes with the analog *α*‐olefin catalyzed by Cp*TiMe_2_(O‐2,6‐^*i*^Pr_2_C_6_H_3_) [124].

In 2020, a series of bottlebrush copolymers with unsaturated side chains have been synthesized through the copolymerization of *α*,*ω*‐dienes (1,9‐decadiene, 1,11‐dodecadiene, and 1,13‐tetradecadiene) with the analog *α*‐olefins (1‐decene, 1‐dodecene, and 1‐tetradecene) using Cp*TiMe_2_(O‐2,6‐^*i*^Pr_2_C_6_H_3_) as precatalyst in combination with [Ph_3_C][B(C_6_F_5_)_4_] and Al^*i*^Bu_3_/Al(*n*‐C_8_H_17_)_3_ (Figure 21). These copolymerizations proceed without cyclization in a quasi‐living manner in *n*‐hexane at subambient temperatures (−30°C/−50°C) and afford ultrahigh–MW copolymers with narrow molecular weight distribution. Diene incorporation ranges from 5.6 to 9.8 mol% for C10 copolymers, 6.6–7.9 mol% for C12, and 3.2–4.5 mol% for C14, with overall diene conversion below 20% [124].

Finally, very recently, Talarico, De Rosa et al. have assembled the pieces of a complex puzzle, establishing, through DFT calculations, the relationships between diastereoselectivity and catalyst structure. Their study encompasses Group IV metallocenes with varying symmetries (*C*_2_, *C*_2V_, and *C*_s_), as well as non‐metallocene Group IV precatalysts, in close analogy with the models proposed for the origin of stereoselectivity in propylene polymerization. The key factors governing the cyclopolymerization of *α*,*ω*‐dienes have thus been elucidated, effectively closing the loop between cyclopolymerization diastereoselectivity and *α*‐olefin polymerization enantioselectivity. For instance, DFT calculations unveil the lower activation energy for the *trans*‐cyclization of 1,5‐hexadiene and an enhanced stability of the *trans*‐chair transition state (TS) compared to other calculated TSs running over the cyclization reaction. These factors account for the 1,5‐hexadiene *trans*‐selectivity observed for the large majority of Group IV (pre)catalysts. The pyridyl(amido) Hf complex stands as the sole exception due to the computed higher stability of the *cis*‐boat TS (Figure 8a), along with the lower activation energy for the formation of *cis*‐MCP units [125].

### 3.4 Beyond Group 4 Metal Catalysts

In contrast to Group IV (pre)catalysts, *α*,*ω*‐diene (co)polymerization using rare‐earth (Group 3 and lanthanides) and late transition metal catalysts is far less explored. Yet, the examples discussed below demonstrate that these untapped catalytic systems enable access to unique polymers and uncover alternative mechanistic paths. Rare‐earth metal catalysts exhibit distinct chemical and physical properties, with metal–carbon and metal–hydride bonds typically extremely reactive [126, 127]. So far, the scope of rare‐earth catalysts has been largely limited to ethylene and polar monomers (alkyl acrylates and lactones) copolymerization. In contrast, only a few examples have been reported for the (co)polymerization of *α*,*ω*‐dienes. Earlier studies on the polymerization of 1,5‐hexadiene and 1,6‐heptadiene date back to 1994 [128], but significant advances were not achieved until 2011–2013, primarily driven by three pivotal studies by Hou et al. [129, 130, 131], yet previously reviewed by Pasini and Takeuchi [1]. Likewise, late transition metal catalysts have been scarcely explored, despite their distinctive regioselectivity in olefin polymerization [132, 133], and the unconventional chain‐walking mechanism (i.e., the catalytically active species jump up and down the growing chain instead of remaining at the chain end) of Ni‐ and Pd‐based catalysts [48, 134].

#### 3.4.1 Polymerization of *α*,*ω*‐Dienes Using Rare‐Earth Catalysts

**FIGURE 22:**
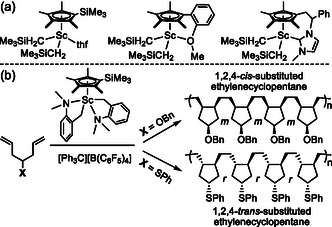
(a) Half‐sandwich Sc catalysts structurally analogous to **CGC** and (b) cyclopolymerization of ether‐ and thioether‐functionalized 1,6‐heptadienes.

Earlier studies on the polymerization of hydrocarbon *α*,*ω*‐dienes using rare‐earth catalysts primarily focused on half‐sandwich Sc catalysts structurally analogous to **CGC** (Figure 22a) and substituted *α*,*ω*‐dienes [1]. While beyond the scope of this review, in 2019 Hou et al. reported the first regioselective, diastereoselective, and stereoselective cyclopolymerization of ether‐ and thioether‐functionalized 1,6‐heptadienes to access functionalized cyclopolymers by a half‐sandwich Sc catalyst (Figure 22b) [135].

**FIGURE 23:**
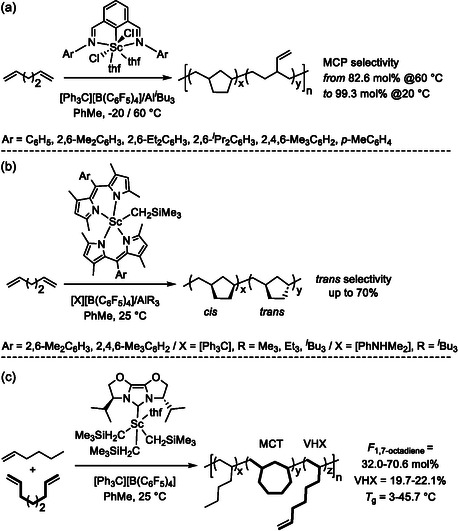
(a) Polymerization of 1,5‐hexadiene catalyzed by Sc complexes with aldimine NCN ligands [136], (b) Polymerization of 1,5‐hexadiene catalyzed by dipyrromethene Sc complexes [138], and (c) Copolymerization of 1,7‐octadiene with 1‐hexene catalyzed by bis(oxazoline) N‐heterocyclic carbene–ligated Sc complex [137].

In contrast to cyclopentadienyl ligands, recent studies have explored the use of nitrogen ligands, encompassing aryldiimine NCN [136], bis(oxazoline)‐derived N‐heterocyclic carbene [137], and dipyrrolidenes [138]. Gao et al. synthesized a series of Sc and Ga complexes bearing aldimine or ketimine NCN pincer ligands with varying aryl substituents. Upon activation with alkylaluminum and trityl borate, only Sc catalysts promote the polymerization of 1,5‐hexadiene with 64% yield in 24 h. The resulting polymers are largely composed of MCP units (up to 99.3%), with only minor incorporation of uncyclized VTM repeating units (Figure 23a ‐ details on the *cis*/*trans* selectivity are not available) [136]. Cyclopolymers with *trans*‐fused MCP rings (*trans* selectivity from 57% to 70%) were obtained using a series of dipyrromethene Sc complexes with almost quantitative yield in 12 h (Figure 23b) [138].

The homopolymerization of 1,7‐octadiene and copolymerization with 1‐hexene was investigated using a bis(oxazoline) N‐heterocyclic carbene–ligated Sc complex in combination with 2 equivalents of [Ph_3_C][B(C_6_F_5_)_4_] [137]. Remarkably, the polymerization of 1,7‐octadiene proceeded with exceptional rate with 95% yield in 5 min, but the resulting polymer was insoluble due to extensive cross‐linking. In contrast, the copolymerization of 1,7‐octadiene with 1‐hexene gave copolymers with high diene incorporation (*F*_1,7‐octadiene_ from 32.0 to 70.6 mol%) and less than 5% cross‐linked fraction (Figure 23c). The copolymers exhibited *T*_g_s from 3°C to 45.7°C due to a significant incorporation of 7‐membered cyclic units (MCT), which restrict chain mobility.

#### 3.4.2 Polymerization of *α*,*ω*‐Dienes Using Fe‐, Ni‐, and V‐Based Catalysts

**FIGURE 24:**
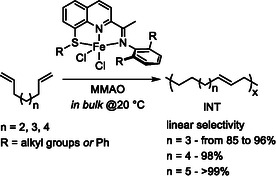
Polymerization of C8–C10 *α*,*ω*‐dienes catalyzed by thioiminoquinoline Fe dichloride precatalysts [60].

Iron is the most Earth abundant, less toxic, and less expensive transition metal and it is considered by the regulatory authorities a “metal with minimum safety concern”. Iron is located in the center of the d‐block: it is “late” and “early” at the same time and should therefore be able to encompass a truly wide range of different chemistries [139, 140]. As such, it is potentially valuable for the synthesis of catalysts employed in high‐volume chemical transformations [141, 142]. Nevertheless, iron catalysis is still in its infancy relative to its well‐established noble metal cousins. In the last decade, substantial progress has been made in iron‐catalyzed polymerization of 1,3‐dienes [143, 144, 145, 146], while the polymerization of non‐conjugated dienes is still largely underexplored. To the best of our knowledge, Osakada et al. in 2007 and 2009 first reported the polymerization of 1,6‐heptadiene using bis(imino)pyridine Fe(II) based catalysts [147, 148]. These iron catalysts promote the cyclopolymerization of 1,6‐heptadiene with over 70% conversion in 5 h and complete selectivity for the formation of *cis‐*fused rings. Following promising early studies, a key study was reported in 2020 by Liu, Guan, Huang et al. in *ACS Catalysis* [60]. They reported the polymerization of C8–C10 *α*,*ω*‐dienes using a series of thioiminoquinoline Fe(II) dichloride precatalysts (Figure 24), which selectivity convert 1,7‐octadiene, 1,8‐nonadiene and 1,9‐decadiene into unsaturated oligomers with nearly exclusive C=C bonds in the main chain via “double‐linear insertion” mechanism (INT unit, see also Figure 7 ‐ path C). This enchained path is dominant for C9 and C10 dienes (selectivity up to 99%), while for C8 it accounts for 85%–96% of the repeating units, with the residual consisting mostly of 6‐membered ring. Similarly, the Fe catalysts mediate the copolymerization of *α*,*ω*‐dienes with ethylene and afford low–MW copolymers with moderate diene incorporation (up to 19 mol%), while maintaining high linear selectivity (up to 99%). DFT calculations reveal that such linear enchainment involved 2,1‐insertion of the first vinyl group into a Fe—C bond and *β*‐H elimination, followed by 1,2‐insertion of the second vinyl group into a Fe—H bond as sketched in Figure 7.

**FIGURE 25:**
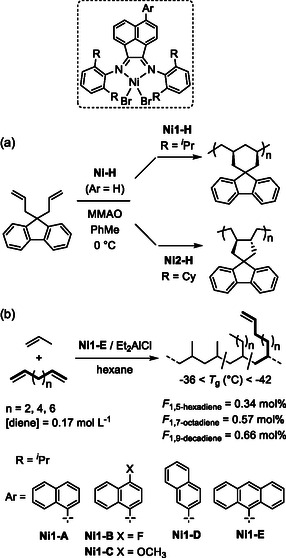
(a) Stereoselective cyclopolymerization of 9,9‐diallylfluorene catalyzed by *α*‐dimmine Ni precatalysts [151] and (b) Copolymerization of C6, C8, and C10 *α*,*ω*‐dienes with propylene catalyzed by *α*‐diimine Ni complexes with congested cyclopolyarene moieties [152].

The use of Ni catalysts to form branched and hyperbranched polymers from 1‐alkanes has been addressed in the past [149, 150], whereas examples of the polymerization of linear *α*,*ω*‐dienes are rare [1]. The most impactful example was reported by Osakada et al. in 2009 [151]. They reported the stereoselective cyclopolymerization of 9,9‐diallylfluorene mediated by *α*‐dimmine Ni complexes. The polymers contain 5‐ and/or 6‐membered rings, depending on the diimine ligand. The Ni complex with *N*−2,6‐diisopropylphenyl substituents (**Ni1‐H**) affords the polymer with *cis*−1,3‐cyclohexylene groups, whereas that with *N*−2,6‐dicyclohexylphenyl substituents (**Ni2‐H**) affords the polymer with *trans*−1,2‐cyclopentylene groups (*T*_g_ up to 209°C) (Figure 25a). More recently, analog Ni complexes were shown to promote the copolymerization of 1,7‐octadiene with 1‐hexene [153], and the copolymerization of ethylene with 1,9‐decadiene and 6‐ethylundeca‐1,10‐diene [154]. The resulting copolymers contain different enchained units (i.e., various saturated alkyl branches) due to the intricate ethylene chain‐walking mechanism and low content of pendant vinyls (from 0.25–1.0 mol%). At 1,9‐decadiene concentrations ≥0.04 mol L^−1^ extensive cross‐linking occurred, whereas no cross‐linking was observed for 6‐ethylundeca‐1,10‐diene under the same conditions.

Very recently, Zhao and Liu reported the copolymerization of C6, C8, and C10 *α*,*ω*‐dienes with propylene using a series of *α*‐diimine Ni complexes bearing sterically congested cyclopolyarene moieties (Figure 25b) [152]. Activated with Et_2_AlCl, the Ni catalysts afford hyperbranched, amorphous copolymers with *T*_g_s from −36°C to −42°C. The polymers are composed of methyl, ethyl, longer than C6 branches, and branches‐on‐branches due to fast and intricate chain walking. The copolymers incorporate a low diene content, that is, 1,5‐hexadiene (0.34 mol%) < 1,7‐octadiene (0.57) < 1,9‐decadiene (0.66), under highly diluted conditions to suppress cross‐linking and ensure gel‐free materials ([diene] = 0.17 mol L^−1^). Increasing the monomer feed concentration to 0.68 mol L^−1^ raises the incorporation of 1,7‐octadiene up to 2.5 mol%, while significantly decreasing the polymer molecular weight. No substantial differences were distinguished among the Ni precatalysts in copolymerization. Notably, the diene does not undergo cyclization.

**FIGURE 26:**
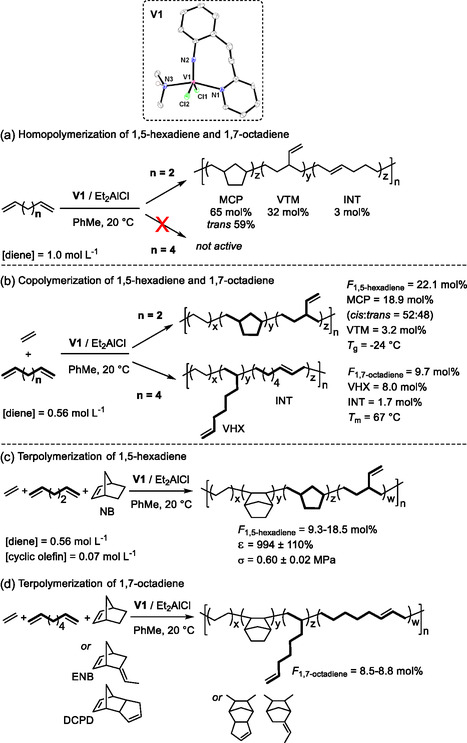
(a) Homopolymerization of 1,5‐hexadiene and 1,7‐octadiene, (b) copolymerization with ethylene [61], (c) Terpolymerization of 1,5‐hexadiene and (d) 1,7‐octadiene with ethylene and various cyclic olefins catalyzed by **V1** [61, 155].

The capability of generating high–MW polymers from *α*,*ω*‐dienes has been recently extended to Group 5 catalysts. In 2021, Leone et al. reported a systematic study on the homopolymerization of 1,5‐hexadiene and 1,7‐octadiene catalyzed by a chelated imido V(IV) precatalyst (hereafter named **V1** ‐ Figure 26) [61]. A series of gel‐free polymers with C=C bonds in the side and main chains have been successfully synthesized only from 1,5‐hexadiene. Indeed, monomer size strongly dictates polymerizability: 1,5‐hexadiene homopolymerizes with 27% yield in 5 h, yielding predominantly MCP units (65.0 mol%) in *trans* configuration (59%), whereas 1,7‐octadiene does not. To further probe their reactivity, copolymerization with ethylene was investigated (Figure 26a). Monomer size also strongly dictates incorporation, productivity, and selectivity (ring closure *vs* ring opening). Copolymerization of ethylene with 1,5‐hexadiene yields high–MW amorphous copolymers (*T*_g_ = −24°C) in which the diene is incorporated mostly as MCP units (18.9 mol%; *cis*/*trans* = 52/48), in addition to a minor fraction of VTM enchained units (3.2 mol%) (Figure 26b). Notably, although 1,7‐octadiene does not homopolymerize, it is efficiently incorporated (up to 9.7 mol%) under copolymerization conditions, affording soluble, semicrystalline copolymers with molecular weight as high as 190 × 10^3^ g mol^−1^. The copolymerization proceeds without intramolecular cyclization, and 1,7‐octadiene is incorporated predominantly as pendant vinyl C6 branches (VHX = 8.0 mol%), in addition to a minor fraction of linear C8 segments with an internal *trans* C=C bond (INT = 1.7 mol%) (Figure 26b).

**V1** precatalyst was also effective for terpolymerization of 1,5‐hexadiene and 1,7‐octadiene with ethylene and various cyclic olefins [i.e., norbornene (NB), 5‐ethylidene‐2‐norbornene (ENB), and dicyclopentadiene (DCPD) ‐ (Figure 26c,d)]. By carefully optimizing the polymerization conditions, a series of gel‐free, high–MW (*M*_w_ up to 200 × 10^3^ g mol^−1^) unsaturated terpolymers were synthesized. The extent of cyclic olefin incorporation follows the order NB > ENB > DCPD (12.4/12.0, 8.4/5.6, and 5.8/4.9 mol% for terpolymerizations with 1,5‐hexadiene and 1,7‐octadiene, respectively), a trend consistent with increasing comonomer steric demand. 1,5‐Hexadiene is incorporated as MCP (6.1–14.2 mol%) and VTM (3.0–4.3 mol%) units, while 1,7‐octadiene is incorporated as VHX (6.0–7.2 mol%) and INT (1.6–2.3 mol%) units. Terpolymers with 1,5‐hexadiene exhibit higher *T*_g_s than those with 1,7‐octadiene (*T*_g_ up to −30°C), as longer flexible branches in the latter prevent efficient chain packing. In contrast, while the terpolymers with 1,7‐octadiene do not readily form stable films (the films tend to curl), those with 1,5‐hexadiene form stable films. Preliminary mechanical studies show that theterpolymers exhibit a ductile behavior, characterized by a well‐defined yield point, an extended drawing plateau, and a final strain‐hardening region prior to fracture. Overall, they behave as soft, flexible plastics with very low stress at break (*σ* = 0.60 ± 0.02 MPa), high stretchability (*ε* = 994 ± 110%), and pronounced permanent deformation in response to the applied load [155].

Prior to 2021, only two back‐to‐back reports by Soga et al. (dating back to 1989) described the (co)polymerization of *α*,*ω*‐dienes using a Group V catalyst (V(acac)_3_/Et_2_AlCl) [156, 157], despite the extensive use of V catalysts in olefin (co)polymerization [158, 159, 160, 161]. All in all, as also observed for Fe and Ni, V precatalysts are underexplored in *α*,*ω*‐diene (co)polymerization. Unlocking their full potential will require the development of original organoligands to access *α*,*ω*‐diene (co)polymers in good yields and with tailored microstructures and properties. More broadly, the use of abundant, less‐toxic and cost‐effective transition metals will foreseeably play a key role in future diene polymerization chemistry.

## 4 Post‐Polymerization Modification: From Graft Copolymers to Dynamically Cross‐Linked Elastomers

Unsaturated C=C motifs provide chemical handles for exploring various chemical strategies for post‐polymerization modification. Alkene is perhaps the most versatile latent functional group to undergo further cross‐linking or functionalization [162, 163, 164, 165, 166, 167]. Post‐polymerization modification is a common approach to differentiate the properties of virgin polyolefins [168]; polymer miscibility or properties such as adhesion and surface tension can be drastically adjusted by the addition of only a small amount of functionality [169, 170]. Moreover, the incorporation of stimuli‐triggered or dynamic, sacrificial covalent or non‐covalent cross‐links (i.e., H‐bonds, ionic interactions, π–π interactions, host–guest complexation, boronic ester, and metal–ligand coordination) have been recently employed to design adaptive bonds via post‐polymerization modification. These dynamically cross‐linked materials behave like traditional thermosets under certain conditions, yet their network topology repeatedly recover malleability upon activation of dynamic exchange interactions. This enable them to overcome the typical trade‐off between robustness and stretchability, as well as between mechanical performance and (re)processability, which are often mutually exclusive in elastomers [171, 172]. A key challenge in post‐polymerization modification of polyolefins is preserving the parent material's properties while avoiding deleterious side reactions, such as C—C bond cleavage or cross‐linking, that compromise tensile performance. Numerous functionalization reactions have been explored, and their mechanisms are generally well understood and largely substrate‐independent. Accordingly, this section focuses on the most promising strategies to functionalize the hydrocarbon polymers discussed herein, with the goal of enhancing their value, particularly in terms of recyclability and reuse.

**FIGURE 27:**
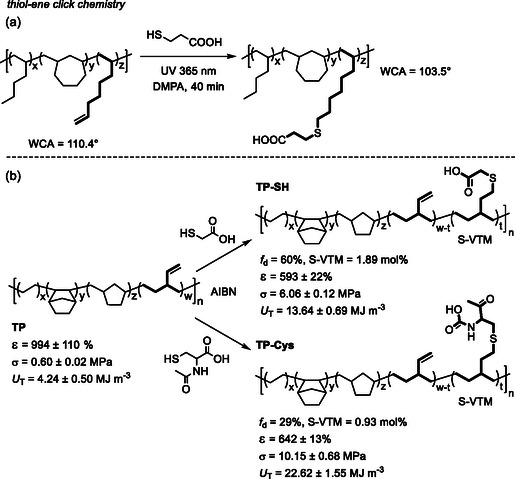
(a) Installation of 3‐mercaptopropionic acid into a copolymer from 1,7‐octadiene and 1‐hexene via UV light [137], and (b) Schematic illustration of post‐polymerization modification of **TP** via thiol‐ene addition (*fd* = functionalization degree) [155].

The most common post‐polymerization functionalizations can be grouped into five major categories: (i) thiol–ene additions, (ii) hydroboration, (iii) hydrosilylation, (iv) grafting and (v) cross‐metathesis. Thiol‐ene click chemistry, typically mediated by radical initiators such as 2,20‐azobis(isobutyronitrile) (AIBN) or triggered photochemically, is rapidly gaining popularity as robust methods. These technologies benefit from quantitative conversions, fast kinetics, air and moisture insensitiveness, and ready commercial availability of diverse thiols [173, 174, 175, 176]. He et al. reported the installation of 3‐mercaptopropionic acid into a copolymer from 1,7‐octadiene and 1‐hexene (double bond = 21.4 mol%). Upon irradiation with 365 nm UV light in the presence of 2,2‐dimethoxy‐2‐phenylacetophenone (DMPA) as the photosensitizer, a carboxyl‐functionalized polyolefin was successfully synthesized (Figure 27a). The introduction of carboxyl moieties proves beneficial for improving the surface hydrophilicity of the otherwise hydrophobic polyolefin; the static water contact angle (WCA) decreases from 110.4° for unfunctionalized polymer to 103.5° [137].

Leone et al. recently demonstrated the utility of thiol‐ene addition for the synthesis of dynamically cross‐linked 1,5‐hexadiene terpolymers (**TP** in Figure 27b) [61, 155]. Thioglycolic acid (**SH**) and *N*‐acetyl‐L‐cysteine (**Cys**) were chosen to introduce H‐bonding motifs, through hydroxyl groups in the former and both hydroxyl and amide functionalities in the latter. Thiol addition was carried out at 100°C using a catalytic amount of AIBN (Figure 27b). The resulting **TP‐SH** and **TP‐Cys** exhibit remarkable strain‐hardening at the late stage of deformation and significantly improved fracture strength and toughness. Toughness was improved by 5 times for **TP‐Cys** (*U*_T_ = 22.62 ± 1.55 MJ m^−3^) and 3 times for **TP‐SH** (*U*_T_ = 13.64 ± 0.69 MJ m^−3^) compared to pristine soft, flexible plastic **TP** (*U*_T_ = 4.24 ± 0.50 MJ m^−3^; *σ* = 0.60 ± 0.02 MPa). The functionalized polymers are tough, yet soft elastomers, whose tensile and elastic properties are dictated by the H‐bond density and the efficiency of the energy dissipation mechanism. At large deformation, the polymers simultaneously dissipate vast energy and maintain excellent elastic recovery after being stretched several times. At low strain, the polymers behave as elastics, exhibiting negligible hysteresis, that is, few dynamic H‐bonds break per cycle, and delocalize the stress concentration to withstand load and to delay premature fracture. Remarkably, the dynamically cross‐linked polymers demonstrate high thermal stability and good (re)processability with no fall in properties for recycling and reuse after being hot melted at least twice.

**FIGURE 28:**
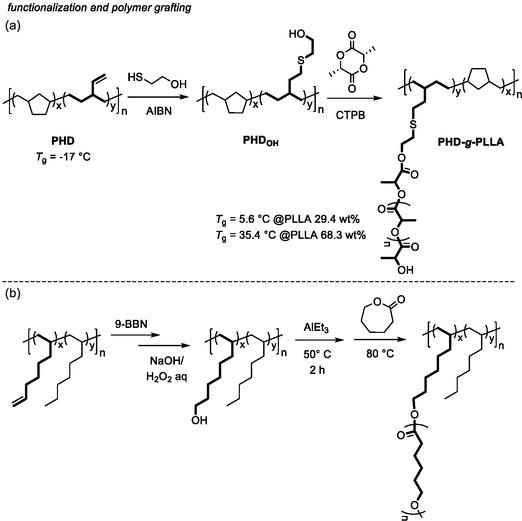
(a) Functionalization and grafting of a polyolefin by ROP of L‐lactide [116] and (b) functionalization and grafting of a 1,7‐octadiene/1‐octene copolymer [177].

In 2025, Liu et al. demonstrated that thiol–ene addition is a convenient strategy to install functional groups that act as initiation sites for the growth of a second polymer chain [116]. Specifically, starting from a polymer derived from 1,5‐hexadiene and containing MCP and VTM units (**PHD** in Figure 28a), functionalization was first carried out with *β*‐mercaptoethanol using AIBN as a radical initiator. The reaction affords a hydroxyl‐functionalized polyolefin (**PHD**_**OH**_). In the subsequent step, **PHD**_**OH**_ (OH = 10.4 mol%) acts as the initiator and, in combination with the cyclic trimeric phosphazene base (CTPB) as the organocatalyst, catalyzed the ring‐opening polymerization (ROP) of *L*‐lactide to yield a graft **PHD‐*g*‐PLLA** polymer (Figure 28a). **PHD‐*g*‐PLLA**, when blended with commercial PLLA, acts as an effective toughening agent while preserving high transparency, strength, and modulus. This is enabled by its uniform dispersion and good interfacial compatibility, which promotes efficient energy dissipation during stretching. A similar strategy was applied by Nomura et al. [177]. Poly(1‐octene‐*co*−1,7‐octadiene) was treated with 9‐borabicyclo [3.3.1]nonane (9‐BBN) and NaOH/H_2_O_2_ (aq) to afford a hydroxyl‐functionalized polymer. This intermediate was then reacted with AlEt_3_ and mixed with ε‐caprolactone, yielding the corresponding graft copolymer vi*a* Al–alkoxide initiated ROP (Figure 28b).

**FIGURE 29:**
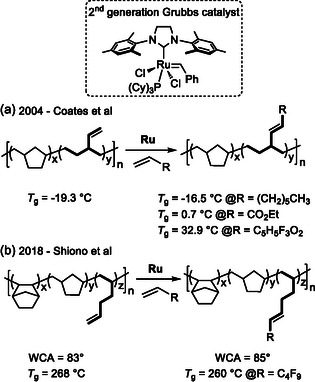
Cross‐metathesis functionalization of polyolefins.

One of the earliest and most inspiring studies on the post‐polymerization functionalization of *α*,*ω*‐diene polymers dates back to 2004. Coates et al. explored cross‐metathesis of a range of commercially available alkenes and acrylates catalyzed by Grubbs’ Ru carbene complex to install various functional groups onto polymers and block copolymers from 1,5‐hexadiene (Figure 29a) [162]. Further, cross‐metathesis was successfully applied by Shiono et al. to a 1,5‐hexadiene/norbornene copolymer (vinyl content = 1.4 mol%) using perfluorobutiletilene and 2^nd^ generation Grubbs catalyst (Figure 29b) [178]. The fluorinated cyclic olefin copolymers have a *T*_g_ of 260°C, while films cast from chloroform show a water contact angle of 85°, only slightly higher than that of the non‐fluorinated precursor (83°).

**FIGURE 30:**
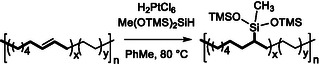
Pt‐catalyzed hydrosilylation of unsaturated linear copolymers [60].

Alternatively, hydrosilylation is a strategy to functionalize unsaturated polymers, although it is less explored due to its reliance on precious Pt catalysts [179]. Post‐polymerization hydrosylilation of *α*,*ω*‐diene polymers dates back to 2000. Uozumi et al. synthesized a series of 1,9‐decadiene/ethylene copolymers which were then grafted with polysiloxane. The hydrosilylation was performed in toluene at room temperature in the presence of a catalytic amount of divinyl tetramethyl disilane Pt catalyst [180]. In 2020, Huang [60] and Ahmadjo [153] demonstrated the Pt‐catalyzed hydrosilylation of unsaturated polyolefins using H_2_PtCl_6_. This approach yields Si‐functionalized materials under relatively mild conditions (80°C–90°C, 15–24 h). In particular, Huang et al. synthesized Si‐functionalized PEs using Me(OTMS)_2_SiH (Figure 30).

## 5 Acyclic Diene Metathesis (ADMET) Polymerization

### 5.1 Mechanism and Historical Overview

**FIGURE 31:**
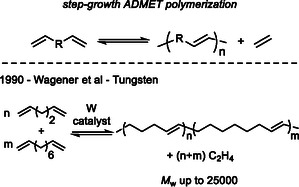
The ADMET reaction (up) and ADMET copolymerization of 1,5‐hexadiene with 1,9‐decadiene (bottom) [181, 182].

ADMET is a step‐growth polymerization driven by the release of a condensate, usually ethylene. ADMET typically yields strictly linear polymers with unsaturated PE‐mimic backbone, as shown in (Figure 31). The accepted ADMET mechanism is well established [2, 3]. The cycle begins with coordination of the *α*,*ω*‐diene to the metal center, followed by formation of a metallacyclobutane intermediate. Its productive cleavage generates the active alkylidene species, which reacts with a diene double bond to form a new metallacyclobutane, driving chain growth. The cycle proceeds through successive coordination of additional diene units or growing polymer chains, with ethylene release as a byproduct.

Initial attempts to apply metathesis to *α*,*ω*‐dienes in the early 1970s exploited nitrosyl Mo and W compounds paired with organoaluminum halides [14]. However, these Lewis acid‐based systems afford only oligomers from 1,5‐hexadiene and 1,7‐octadiene due to competing olefin alkylation and acid‐catalyzed vinyl addition side reactions [183]. Later, Schrock et al. developed a series of highly reactive neutral Mo [184] and W [185, 186] metathesis catalysts. The use of these Lewis acid free catalysts by Wagener's group led to the first quantitative metathesis polymerization [181], and copolymerization of 1,5‐hexadiene and 1,9‐decadiene (Figure 31) [182]. The resulting polymers behave like PE mechanically, but the backbone unsaturation turns them into a chemically “editable” PE platform, enabling smart applications. These pioneering studies first produced high–MW polymers at low catalyst loadings, establishing ADMET as a synthetic platform to yield diverse macromolecular structures.

In particular, ADMET allows access to precision PEs with methyl, ethyl or hexyl branches that closely mimic polymers obtained via insertion chain‐growth polymerization. However, one key advantage of ADMET is that it avoids the random nature of branching (and length) in PE by introducing only and exactly the branch identity targeted, and by placing the desired alkyl branch exactly in a specific place along the main chain, a direct consequence of its step‐growth mechanism. In contrast, a key limitation from a scalability and practical standpoint lies in the synthesis of *α*,*ω*‐dienes with symmetrically positioned alkyl branches. This step can still signify a bottleneck in large‐scale applications. Notably, however, recent methodologies have streamlined the process: a simple two‐step sequence ‐ alkylation and decyanation ‐ affords the preparation of a wide range of hydrocarbon *α*,*ω*‐dienes in nearly quantitative yields. These monomers can then be efficiently polymerized to afford materials that closely mimic commercial LLDPEs. Readers are guided to a recent review on the area for a more depth overview [6].

### 5.2 Functional *α*,*ω*‐Dienes as Molecular “Lego” Blocks ‐ Selected Examples

**FIGURE 32:**
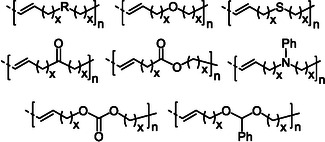
Some examples of polymers made by ADMET.

ADMET has been extensively exploited to synthesize a variety of polymers, block and graft copolymers, synthetically inaccessible by any other techniques. Figure 32 shows only some representative examples of the polymers produced from hydrocarbons and *α*,*ω*‐dienes containing linkages such as ethers [187], esters [188], carbonate esters [189], thioethers [190], amines [191], silanes and siloxanes [192], sulfonic esters [193], and phosphoesters [194]. The integration of these diverse motifs has enabled the creation of polymers with precisely defined architectures, unprecedented regioregularity and unique properties. Remarkably, *α*,*ω*‐dienes act as molecular “Lego” blocks, offering a modular and programmable blueprint for polymers with otherwise inaccessible functionalities and strategic placed cleavable sites. This enable facile depolymerization and predictable end‐of‐life behavior [195]. Collectively, *α*,*ω*‐dienes in which the two vinyls are covalently linked by a functional “tether”, which can be appropriately designed in order to imprint elements of chemical information into the polymer main chain have transformed non‐conjugated dienes from a historically overlooked monomers into a versatile scaffold at the intersection of polymer engineering and depolymerization [8, 48].

**FIGURE 33:**
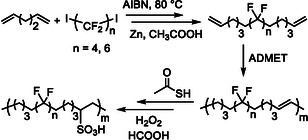
Synthesis of precisely sulfonated polymers periodically placed perfluoroalkyl segments and sulfonic acid groups.

However, while more recently ADMET of unactivated hydrocarbon *α*,*ω*‐dienes has received comparatively limited attention, hydrocarbon non‐conjugated dienes continue to play an important role as precursors or, as highlighted by Li et al. as “*chemically dormant*” substrates in atom transfer radical addition (ATRA) to access a range of functionalized dienes while suppressing undesired side reactions. Indeed, ATRA transformation of simple dienes such as 1,5‐hexadiene and 1,7‐octadiene affords *α*,*ω*‐diene derivatives, which in turn yield, via ADMET, periodic and precision homopolymers or copolymers to disrupt homopolymer periodicity and limit regular chain packing. For example, the group of prof. Li have successfully enlarge the scope of 1,5‐hexadiene and/or 1,7‐octadiene to monomers containing perfluoroalkyl segments [–(CF_2_)_*n*_–, *n* = 4, 6] (Figure 33) [196] γ‐butyrolactone [197] or dibromohexanedioate units [198]. Via ADMET, these monomers have been transformed into precision polymers incorporating polar moieties and internal C=C bonds (Figure 33). These unsaturations have subsequently been converted into sulfonic acid groups through post‐modification. This transformation affords semi‐crystalline sulfonated PEs, which may be suitable for efficient proton transport membrane applications [199, 200].

All in all, *α*,*ω*‐dienes are versatile platforms for the construction of monomers and polymers that mimic the structure and function of sophisticated natural examples of high structural complexity and functions. Their tunable composition also enables advanced strategies for controlled plastic degradation, opening new opportunities for the design of sustainable polymeric materials. ADMET provides promising strategies for the synthesis and recycling of degradable PE‐like materials, but it is still constrained by key challenges, including high catalyst cost, accidental polymer degradation, and low mechanical strength.

## 6 The Synthesis of Rings

### 6.1 Cyclization and Cycloisomerization

The synthesis of rings is central to organic synthesis. Cyclic compounds are ubiquitous in chemistry, from strained 3‐membered rings to large macrocycles. The chemist has two common strategies to synthesize a ring: annulation or cyclization. In an annulation two separate reactive entities combine to create a ring by the formation of two new bonds, while cyclization involves the formation of a ring by the reaction of two ends of a linear sequence, forming a single bond. Cycloisomerization comprise a particularly efficient subset of this class, in which an unsaturated linear substrate rearranges intramolecularly to generate a ring without a net change in molecular formula [201]. Cyclization and cycloisomerization of *α*,*ω*‐dienes have figured prominently as key synthetic reactions for the enantioselective construction of carbocycles and heterocycles.

**FIGURE 34:**
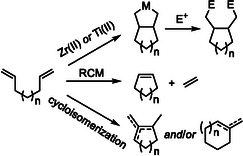
Examples of cyclization of hydrocarbon *α*,*ω*‐dienes.

Cyclization of *α*,*ω*‐dienes promoted by low‐valent Ti or Zr reagents have been extensively investigated (Figure 34). This method enables the synthesis of a variety of functionalized cyclic products by trapping metallacyclopentane intermediates with various electrophiles (E^+^). Nevertheless, it is limited by the need for stoichiometric amounts of metal, which is a critical drawback. RCM of *α*,*ω*‐dienes has opened a new path for the construction of cyclic products; however, RCM is not optimal in terms of atom economy, as ethylene is lost during the cyclization process. In contrast, cycloisomerization of *α*,*ω*‐dienes is an atom‐efficient reaction in which C—C bond formation is achieved with concomitant transposition of a hydrogen atom, without the need for additional reagents [5, 202].

**FIGURE 35:**
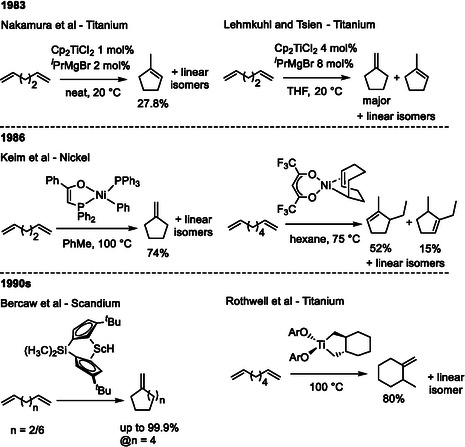
Selected examples of cyclic fine chemicals derived from diene cyclization.

Progress in cyclization and cycloisomerization of *α*,*ω*‐dienes has been driven by the development of increasingly selective and versatile catalysts, significantly expanded substrate scope and improved isomeric selectivity. Figure 35 illustrates the versatility of *α*,*ω*‐dienes, showcasing the broad range of cyclic fine chemicals that can be accessed. For a more comprehensive overview of *α*,*ω*‐diene cyclization, the review by Yamamoto is highly recommended [5].

**FIGURE 36:**
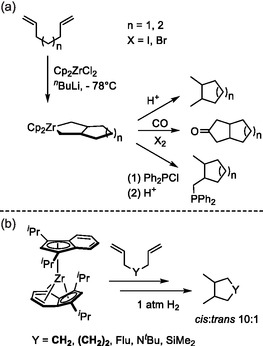
(a) *α*,*ω*‐Diene cyclization promoted by in situ generated Negishi reagent, and (b) cyclization of 1,6‐heptadiene and 1,7‐octadiene with a Zr sandwich complex [207].

Isolable metallacycles are also valuable intermediates to construct fundamental carbocycles and heterocycles in the presence of proton sources, halide sources or carbon monoxide [203, 204, 205, 206]. Figure 36a shows some representative examples of *α*,*ω*‐diene cyclization promoted by the in situ generated Negishi reagent, that is, (η^5^‐C_5_H_5_)_2_Zr. In 2011, Chirik et al. investigated related *α*,*ω*‐diene metallacycles and cyclization chemistry from η^9^,η^5^‐bis(indenyl)zirconium sandwich compound and an *ansa*‐titanocene dinitrogen complex [207]. Treatment of 1,6‐heptadiene and 1,7‐octadiene with the zirconocene leads to rapid cyclometallation, affording the corresponding zirconacyclopentane intermediate as mixtures of *cis* and *trans* diastereomers that gradually isomerize to the thermodynamically favored *trans* form. Upon treatment with aqueous acid or water, or with two equivalents of PhSiH_3_, these metallacycles undergo reductive elimination to release substituted pyrrolidines. Particularly, addition of one atmosphere of dihydrogen to a benzene‐*d*_6_ solution of a *cis*:*trans* mixture (10:1) of zirconacyclopentane intermediate resulted in release of the free 3,4‐dimethyl pyrrolidine as a 10:1 mixture of *cis* and *trans* isomers (Figure 36b). In contrast, the analogous titanocene fail to generate the free cyclic product.

**FIGURE 37:**
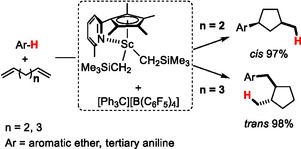
Tandem cyclization/hydroarylation of 1,5‐hexadiene and 1,6‐heptadiene triggered by Sc‐Catalyzed C–H Activation [208].

Besides the formation of carbocyclics via cycloisomerization or diene cyclization combined with carbometallation, *α*,*ω*‐dienes have also been used in cyclizations triggered by C—H activation. Chen and Hou reported that Sc–catalyzed cyclization 1,5‐hexadiene or 1,6‐heptadiene yields 1,3‐disubstituted cyclopentane derivatives (Figure 37) [208]. The reaction, initiated by aromatic C—H activation of pyridines [209], aryl ethers, and anilines [208], and two consecutive carbo‐metallation steps construct the 5‐membered carbocycles.

**FIGURE 38:**
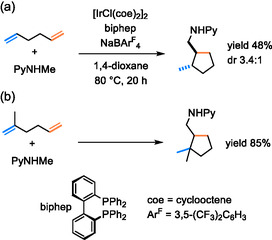
Ir–catalyzed cyclization of (a) 1,5‐hexadiene and (b) 2‐methyl‐1,5‐hexadiene [210].

In 2022, Nishimura et al. investigated the Ir–catalyzed sp^3^ C—H alkylation of an *N*‐methyl group with 1,5‐hexadiene and 2‐methyl‐1,5‐hexadiene [210]. The reaction proceeds via intermolecular alkylation of the *N*‐methyl group with a vinyl moiety and successive intramolecular cyclization to afford the 5‐membered carbocycles in high yield (Figure 38).

**FIGURE 39:**
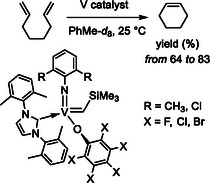
RCM of 1,7‐octadiene using vanadium(V)–alkylidene catalysts [212].

Carbocycles can be synthesized also via RCM using *α*,*ω*‐diene substrates. In the classical view of olefin metathesis, RCM and ADMET are often considered competing paths. However, as stated by Prof. Fogg “*ADMET can be viewed as intrinsic*, *rather than inimical*, *to RCM*.” as many functionalized *α*,*ω*‐dienes (ester, ether, catechol, malonate derivatives) affords oligomers under certain conditions [211]. Within the scope of this review, RCM of simple hydrocarbon *α*,*ω*‐dienes typically leads to the formation of unsaturated rings [4]. As an illustrative example – intended to complement, though not exhaustively – the overview of carbocycles accessible from simple *α*,*ω*‐dienes, it has recently been reported that the RCM of 1,7‐octadiene affords exclusively cyclohexene using NHC‐supported (arylimido)vanadium(V) alkylidene complexes (Figure 39) [212].

### 6.2 Cycloaddition – The Action of Redox‐Active Ligands

Cycloaddition of unactivated dienes, promoted by light, heat, Lewis acids or high pressure are generally inefficient, or harsh conditions are necessary to reach satisfactory yields of cycloadducts. This is largely due to competing dimerization paths, which frequently dominate over the desired cycloaddition [213]. By contrast, metal‐catalyzed cycloadditions of *α*,*ω*‐dienes have emerged as an attractive and atom‐economical approach to strained 4‐membered rings via formal [2 + 2] processes, as metal coordination significantly alters the reactivity of otherwise unactivated substrates. Indeed, metal complexation transiently polarizes and activates the targeted dienes, enabling reactivity patterns that are inaccessible under conventional conditions. Prof. Chirik has extensively investigated cycloaddition of ethylene, *α*‐olefins, and 1,3‐dienes to generate cyclobutanes using Fe and Co based catalysts [214, 215].

**FIGURE 40:**
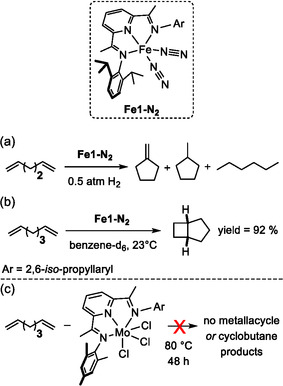
(a) Hydrogenative cyclization and cycloisomerization of 1,5‐hexadiene promoted by **Fe1‐N**_**2**_ and H_2_ [216], (b) cycloaddition of 1,6‐heptadiene promoted by **Fe1‐N**_**2,**_ [217] and (c) failed attempts at Mo‐promoted [2 + 2] cycloaddition of 1,6‐heptadiene [218].

Within the scope of this review, the reactivity of 1,5‐hexadiene, 1,6‐heptadiene and 1,7‐octadiene using bis(imino)pyridine Fe dinitrogen (or dihydrogen) complexes [216, 217], and Mo complexes has been investigated [218]. The reactivity of 1,5‐hexadiene was explored using (^*i*^PrPDI)Fe(N_2_)_2_ (^*i*^PrPDI = 2,6‐(2,6‐*i*Pr_2_C_6_H_3_NCR)_2_C_5_H_3_N; R = Me, Ph – **Fe1‐N**_**2**_). Stirring the diolefin with 10 mol% of iron catalyst under 0.5 atm of H_2_ yields a mixture of methylenecyclopentane, methylcyclopentane and *n*‐hexane (Figure 40a) [216]. Using the iron dihydrogen (^*i*^PrPDI)Fe(*η* ^2^‐H_2_) analog as the precatalyst enhances the reaction selectivity, yielding exclusively methylenecyclopentane. The study was then extended to 1,6‐heptadiene, which, in stark contrast to the C6 diene, yields *n*‐heptane and the *cis* isomer of bicyclo [0.2.3]heptane arising from intramolecular [2π+2π] cycloaddition of the two terminal olefins (Figure 40b) [217]. Computational, spectroscopic, and X‐ray diffraction metrics established an intermediate spin (*S*_Fe_ = 1) ferrous center complexed by the bis(imino)pyridine dianion [L^red^]^2−^.

**FIGURE 41:**
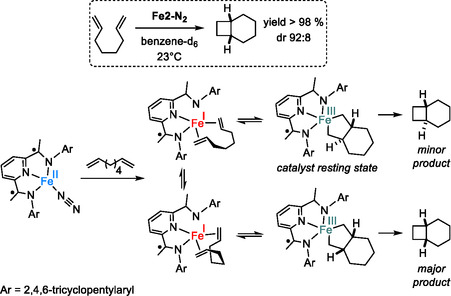
Simplified schematic of the proposed catalytic cycle for the Fe‐catalyzed intramolecular [2 + 2] of 1,7‐octadiene (Chirik et al.) [216].

In the presence of 5 mol% (^tric^PDI)Fe(N_2_) (^tric^PDI = 2,6‐(2,4,6‐tricyclopentyl)‐C_6_H_2_N = CMe)_2_C_5_H_3_N – **Fe2‐N**_**2**_) in benzene‐*d*_6_, 1,7‐octadiene underwent [2π + 2π] cycloaddition to form *cis*‐bicyclo [4.2.0]octane in > 98% conversion and for 92:8 diastereomeric ratio after 9 h at 23°C (Figure 41) [216]. Isolation of catalytically relevant intermediates and single crystals suitable for X‐ray diffraction studies established metrics consistent with the singly reduced form of the chelate ligand [i.e., the monoanionic radical state (L˙)^−^], supporting an Fe(III) oxidation state. A pyridine(diimine) iron *trans* bimetallacycle was identified as the catalyst resting state. Interestingly, dissolution of the iron *trans* bimetallacycle in benzene‐*d*_6_ yields predominantly the *cis* cyclobutane product, establishing interconversion between the *trans* and the *cis* isomers over the catalytic reaction. The exceptionally comprehensive combination of kinetic data, identification of the catalyst resting state, spectroscopy studies and DFT calculations supports a distinctive and unrecognized catalytic cycle, which is presented here in a simplified form in Figure 41. A particularly noteworthy feature of the proposed cycle is that, although the resting state of the intramolecular [2 + 2] cycloaddition is the *trans*‐bimetallacycle, the reaction predominantly affords the corresponding *cis*‐bicycle. This apparent inconsistency has been the subject of additional detailed studies by the authors, and the reader is strongly encouraged to refer to the original article for a comprehensive analysis [216].

To investigate whether reduced pyridine(diimine) Mo complexes were competent to generate strained cyclobutanes similarly to Fe, the reactivity of 1,6‐heptadiene with the pyridine(diimine) MoCl_3_ was investigated [218]. The Mo complex was reduced with excess 1.0% Na(Hg) in the presence of 2 equiv. of 1,6‐heptadiene, yielding a mixture of bis‐olefin complexes that converge to a single species; however, even after optimization of the work‐up, no metallacycle or cyclobutane products were obtained (Figure 40c).

Collectively, these studies highlight how subtle variations in diene structure, ligand substitution, and metal identity (first‐ *vs* second‐row) strongly affect reactivity, selectivity, and practical polymerizability. Such insights provide valuable design levers for future catalyst development. In addition, remarkably, the above data suggested that the key feature of the catalytic cycle using the Fe congeners is the ligand redox activity, that is the bis(imino)pyridine participate in redox chemistry with the metal, rather than existing as spectators. This concerted electron transfer preserves the ferrous oxidation state throughout the entire cycle and may prevent complications from Fe(0) precipitation that are observed with other metallocycles [219]. Similarly, studies by Leone, Groppo et al. have shown that a bidentate iminopyridine ligand serve as electron reservoirs working in concert with Cr ions and providing an unexpected utility in the polymerization of ethylene [220, 221]. Overall, these independent studies suggest, though not conclusively, that the first‐row metal complexes are thus far unique in promoting redistribution of the electron density from the metal ion to the ligand, and that this metal‐to‐ligand synergy plays a key role in catalyst reactivity. However, while ligand redox activity may serve as a reliable predictor of reactivity, it does not fully account for the range of behaviors observed across monomer classes (olefins *vs* diolefins) and organic transformations. Further studies, particularly those extending to second‐ and third‐row metals, may help to clarify these trends and potentially broaden the scope of hydrocarbon non‐conjugated dienes reactivity.

## 7 Miscellaneous

**FIGURE 42:**
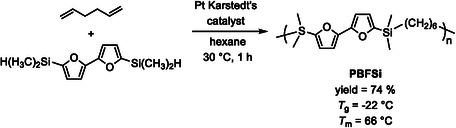
Recyclable poly(bifurancarbosilane) (**PBFSi**) from a biomass‐derived bifuran‐based monomer and 1,5‐hexadiene [224].

Beyond the transformations discussed above, *α*,*ω*‐dienes participate in numerous other organic reactions. Our brief discussion below is not intended to be comprehensive, but rather to highlight representative examples illustrating how the hydrocarbon *α*,*ω*‐dienes have been successfully translated into additional valuable chemicals and intermediates. Notable transformations include:

i. hydroboration of acyclic *α*,*ω*‐dienes which yields cyclic β‐chloroorganoboranes (typically in addition to polymeric products) [222];
ii. cycloboration of C6–C10 *α*,*ω*‐dienes with boron halides in the presence of metallic Mg and Cp_2_TiCl_2_ as the catalyst which yields strained ring 1,2‐disubstituted boriranes [223];
iii. hydrosilylation copolymerization of 1,5‐hexadiene with dihydrosilylbifuran using the Pt Karstedt's catalyst to synthesize a poly(bifurancarbosilane) (**PBFSi** in Figure 42), composed of silicon and bifuran repeating units which find application in electrical devices, heat‐resistant materials and fluorescence sensing coatings [224];
iv. hydroformylation of *α*,*ω*‐dienes mediated by rhodium catalysts which affords aldehydes and linear long chain dialdehydes.

**FIGURE 43:**
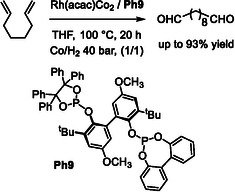
Rh‐catalyzed bis‐hydroformylation of 1,7‐octadiene in the presence of bisphosphite **Ph9** [227].

In particular, hydroformylation of *α*,*ω*‐dienes provides access to key precursors for various pharmaceutical intermediates, cross‐linking agents for protein and polysaccharides as well as decanedioic acid, 1,10‐decanediamine and 1,10‐decanediol, which serve as fundamental precursors in polyamide manufacturing [225]. In the Rh‐catalyzed bishydroformylation of 1,7‐octadiene to decanedial, achieving satisfactory high chemoselectivity and regioselectivity without compromising reactivity is still challenging, primarily due to competing reactions arising from alkene isomerization and hydrogenation. To address these limitations, Breit et al. developed hydroformylation catalysts based on ligands capable of self‐assembly via hydrogen bonding within the coordination sphere of the rhodium center [226]. More recently Wu, Dong et al. employed an unsymmetrically bulky bisphosphite ligand for the Rh‐catalyzed bis‐hydroformylation of 1,5‐hexadiene, 1,7‐octadiene and 1,9‐decadiene to linear dialdehydes (Figure 43). This approach effectively suppresses the undesired isomerization of terminal alkenes to internal alkenes while maintaining high catalytic activity and selectivity [227].

**FIGURE 44:**
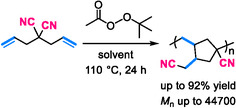
Synthesis of functionalized cyclic olefin polymers via GTRCP [228].

More recently, further derivatization of *α*,*ω*‐dienes has further expanded their utility beyond traditional hydrocarbons. In 2025, Sun, Zhu et al. introduced an unconventional metal‐free radical cyclopolymerization, that is, group transfer radical cyclopolymerization (GTRCP), of non‐conjugated dienes to synthesize cyclic olefin polymers, incorporating a functional cyano group migration process (Figure 44). The obtained polymers demonstrate great potential as robust interphase layer materials in anode‐free Li batteries, opening up new opportunities for practical energy storage applications of cyclic olefin polymers [228].

As discussed throughout this section, hydrocarbon *α*,*ω*‐dienes are highly versatile substrates and (co)monomers for a wide variety of catalytic transformations, attracting sustained interest in both synthetic and polymer chemistry. In several subsections, the discussion was intentionally focused on examples reported since 2018, as earlier contributions had already been comprehensively reviewed by Pasini and Takeuchi [1]. In other cases, however, a broader overview was provided to highlight the diversity and scope of the most relevant transformations. To consolidate the studies presented in this section and facilitate comparison among the different methodologies, Table 2 summarizes a selection of the most representative examples discussed above. Beyond offering a concise overview, the table is intended to serve as a practical reference for identifying suitable dienes and catalysts in relation to specific transformations and target products.

**TABLE 2:** Overview of some of the most representative examples reported from 2018 onward involving the catalytic conversion of *α*,*ω*‐dienes.

Metal	Ligand	*α*,*ω*‐diene	Active for	Product	Potential applications	Ref.
Sc	Aryldiimine NCN dipyrromethene NN	C6	Cyclopolymerization	Optical active polymers	Liquid chromatography	[136, 138]
	Oxazoline NN	C8	Copolymerization with 1‐hexene	High–MW copolymers	Reactive intermediates	[137]
	Cp	C6, C7	Cyclization	5‐membered carbocycles	Chemical intermediates	[208]
Ti	TiCl_4_/MgCl_2_	C6–C10	Copolymerization with ethylene and *α*‐olefins	Cross‐linked polymers	‐ Lightweight packaging ‐ Additives for fluids ‐ Impact modifiers	[42, 93, 94, 95, 96, 97]
	**CGC**	C8	Copolymerization with ethylene	Crystalline copolymers	Electronic packaging applications	[119]
	**CGC**	C10	Terpolymerization with ethylene and 1‐octene	High–MW terpolymers	Encapsulation material for solar cell lamination	[120]
	Cp	C6, C8, C10	Cycloboration	1,2‐disubstituted boriranes	Pharmaceutical agents	[223]
Zr	Cp	C6	Copolymerization with propylene	High–MW copolymers	Thermoplastic elastomers	[102]
Hf	CPAM	C6	Cyclopolymerization	Optical active polymers	Liquid chromatography	[78, 82]
	**Hf1**	C6	(Co)polymerization with olefins	(Block) copolymers	Robust thermoplastic elastomers	[104, 105, 110, 114, 115]
V	NHC‐ arylimido	C8	Ring closing metathesis	Cyclohexene	Chemical intermediates	[212]
	Imido	C6, C8	Terpolymerization with ethylene and cyclic olefins	High–MW polymers	‐ Soft elastomers ‐ Reactive polymer intermediate	[61, 155]
Fe	Iminoquinoline NNS	C8–C10	(Co)polymerization	Unsaturated PEs	‐ Compatibilizers ‐ Reactive intermediates	[60]
	Bis(imino)pyridine NNN	C6–C8	Cycloaddition	Carbocycles	Chemical intermediates	[216, 217]
Ir	Chiral bidentate phosphine	C6	Cyclization	5‐membered carbocycles	Chemical intermediates	[210]
Rh	acac	C6, C8, C10	Hydroformylation	Linear dialdehydes	Polyamide manufacturing	[227]
Ni	Diimine NN	C6, C8, C10	Copolymerization with propylene	Highly branched polyolefins	Elastomers	[152]
Pt	Karstedt's catalyst	C6	Hydrosilylation Copolymerization	PBFSi	‐ Electrical devices ‐ Heat‐resistant materials	[224]

Abbreviations: acac, acetylacetonate; CGC, constrained geometry catalyst; Cp, cyclopendienyl ligand; CPAM, caproamidinate ligand; PBFSi,  poly(bifurancarbosilane).

## 8 Summary and Outlook

Chemical trasformations of *α*,*ω*‐dienes have reached impressive stereoselectivity and diastereoselectivity to access a variety of carbocycles and precision polymers. Such progress builds on recent advances in transition metal (pre)catalysts and fundamental mechanistic insights. However, it still relies on a relatively narrow subset of early and late transition metal catalysts, highlighting both their remarkable versatility and the practical limitations imposed by long‐term sustainability. The cost of catalysts is a critical consideration. Most current strategies rely on precious, noble metals which are much more expensive than the chemicals recovered. Looking ahead, the emergence of alternative, earth‐abundant metals is an intriguing but still early stage frontier. At the same time, redox active ligands may unlock reactivity not accessible through hydrocarbon dienes alone, and the synthesis of otherwise unconventional rings and macromolecules.

The insertion polymerization of acyclic *α*,*ω*‐dienes has attracted growing attention, and the subject is progressing fast. However, the polymerization of linear *α*,*ω*‐dienes presents a fundamental dichotomy: their bifunctional nature grants access to polymers with cyclic enchained units and unsaturated side or main chains, while at the same time facilitate intermolecular cross‐linking and gelation. A major technical hurdle is the requirement for highly dilute conditions to suppress cross‐linking and ensure reliable kinetic data and polymer solubility. Yet, when properly controlled, cross‐linking yields tough, soft elastomers. Future opportunities include the exploration of reactor configurations that alleviate solution viscosity and enhance mass transfer. In addition, absolute control over tacticity and diastereoselectivity – which dictates *cis/trans* configuration of cyclic units – is yet the most key challenge. The competition between ring‐closure and ring‐opening is only one of several potential driving forces for insertion polymerization of *α*,*ω*‐dienes. While ring strain energy offers an intuitive metric for assessing feasibility, it alone does not dictate polymerizability or kinetic. Indeed, other steric effects, conformational stabilization of the catalytically active intermediates, electronic delocalization and intermolecular cross‐linking can each play decisive roles in determining the ease and thermodynamic favorability of chain propagation. The overall driving force for chain growth therefore reflects not only the monomer size but also the stabilization gained in the ring closed intermediate ‐ a balance of geometric and electronic factors that mirrors other chemical transformations. Overall, the examples discussed in this review demonstrate that insertion polymerization of acyclic hydrocarbon *α*,*ω*‐dienes is a powerful approach to access optically active cyclopolymers and unsaturated polymers. Importantly, periodic C=C bonds can be incorporated along the main or side chains, providing sites for selective cleavage or further functionalization. At the same time, LCCTP offer compelling advantages from a sustainability standpoint as the use of targeted CTAs enables the installation of degradable functionalities into the polymer main chain. Looking ahead, we envision that simple *α*,*ω*‐dienes may hold significant promise in the field of elastomers and could, in the long term, compete with natural rubber. At present, natural rubber is the benchmark elastomer due to its unique strain‐induced crystallization, a feature that has not been fully replicated in synthetic alternatives such as styrene‐butadiene rubber or poly(isoprene). However, beyond crystallization, non‐conjugated dienes offer distinct advantages: they can install LCBs as a route to mechanical reinforcement and chemical handles to integrate dynamic cross‐links. Incorporating dynamic, reversible cross‐links may solve the trade‐off between strength and toughness, and irreversible cross‐linking and elasticity.

The past 10 years have seen remarkable advances in the area of cycloisomerization and cycloaddition reactions. A breathtaking array of dienes can now be cyclized with superb selectivity. Historically and to this day, cyclization reactions serve as fundamental testing ground for cutting edge approaches in asymmetric catalysis to yield carbocycles and heterocycles which are ubiquitous structural motifs and key synthetic intermediates for drug discovery and development.

Closely related efforts focus on alternative strategies to bypass traditional petrochemicals and produce hydrocarbon *α*,*ω*‐dienes from renewable sources and plastic waste upcycling. Reducing reliance on fossil resources and harsh reaction conditions would significantly expand the scope of accessible *α*,*ω*‐dienes. By identifying common conceptual approaches, we critically discuss how advantages and challenges of each technology foster sustainable chemical practices and a circular plastic economy. Ethenolysis is attractive from both green chemistry and circularity perspectives, as it enables more efficient, operationally simple, and environmentally benign processes. Nevertheless, for these approaches to become genuinely sustainable, additional considerations related to solvent use, Ru‐based catalysts, and ethylene consumption must be carefully addressed and, where possible, replaced by more sustainable technological alternatives. While the sourcing of *α*,*ω*‐dienes from renewable feedstocks and via plastic recycling offers clear sustainability benefits, these approaches still suffer from limited scalability, stability, and selectivity. Current efforts are directed toward the selective valorization of plastic waste into fine chemicals and polymeric materials with improved recyclability, without compromising performance. However, their relevance to real‐world plastic waste streams remains underdeveloped and will ultimately require technologies capable of handling complex and heterogeneous polymer mixture, as well as catalysts tolerant toward inorganic additives, plasticizers, and other contaminants. Achieving this will likely require fundamentally new technologies and more robust, cost‐effective catalysts even to suppress undesirable self‐metathesis, isomerization side reactions, and long‐term deactivation.

We anticipate that the utility of simple *α*,*ω*‐dienes will continue to grow as their production from renewable sources and plastic waste expands, while exciting challenges lie ahead.

## Funding

This study was supported by Consiglio Nazionale delle Ricerche.

## Conflicts of Interest

The authors declare no conflicts of interest.

## Data Availability

Data sharing not applicable to this article as no datasets were generated or analysed during the current study.
